# Investigation of Cytokine Profiles, the Expression of Genes Involved in Somatic Hypermutation and Peripheral Tolerance, and Their Correlation with Autoantibody Levels in Northern-Thai Immunodeficient Patients Carrying Anti-Interferon-γ Autoantibodies

**DOI:** 10.3390/ijms27156998

**Published:** 2026-08-04

**Authors:** Kritsadee Rattanathammethee, Kriangkrai Chawansuntati, Nongkran Lumjuan, Nattaya Nusartsang, Romanee Chaiwarith, Jutarat Praparattanapan, Khuanchai Supparatpinyo, Jiraprapa Wipasa

**Affiliations:** 1 Research Institute for Health Sciences, Chiang Mai University, Chiang Mai 50202, Thailand; kritsadee.ly@gmail.com (K.R.); kriangkrai.ch@cmu.ac.th (K.C.); nongkran.l@cmu.ac.th (N.L.); nattaya.n@cmu.ac.th (N.N.); khuanchai.s@cmu.ac.th (K.S.); 2 Department of Internal Medicine, Faculty of Medicine, Chiang Mai University, Chiang Mai 50202, Thailand; romanee.c@cmu.ac.th (R.C.); jutarat.p@cmu.ac.th (J.P.)

**Keywords:** cytokines, gene expression, immune deficiency, anti-interferon-γ autoantibodies, autoantibody, intracellular organism infection, adult-onset immunodeficiency

## Abstract

Anti-interferon-γ (IFN-γ) autoantibody (AAb)-associated adult-onset immunodeficiency (AOID) is an emerging disorder in East Asian adults characterized by severe opportunistic infections despite negative HIV status. The mechanisms driving anti-IFN-γ AAb production remain unclear. This study investigated B-cell gene expression, serum cytokine profiles, associations with AAb levels, and IFN-γ genetic variations in AOID patients. A cross-sectional study was conducted in 63 AOID patients and 30 healthy controls at Chiang Mai University Hospital, Thailand. Patients were classified as active or inactive according to infection status. B-cell gene expression, serum cytokines, anti-IFN-γ Aabs levels, and IFN-γ polymorphisms were analyzed by quantitative PCR, multiplex assays, ELISA, and nucleotide sequencing, respectively. AOID patients exhibited increased *PAX5* and *BLIMP1* expression and reduced *XBP1*, *BCL6*, *KRAS*, and *BAFF* expression. Active patients had higher levels of TNF-α, IL-6, IL-17A, IL-10, IL-21, and more chemokines than inactive patients. AAb levels positively correlated with TNF-α, IL-6, and IL-10. Correlation analysis showed positive associations among *PAX5*, *XBP1*, and *KRAS*, and a negative association between *BLIMP1* and *BCL6*. No significant IFN-γ sequence differences were identified within the analyzed transcript region. These findings suggest altered B-cell gene expression and inflammatory cytokine profiles in AOID, which may be associated with the immunological alterations observed in these patients and may provide insights into potential mechanisms underlying anti-IFN-γ AAb production.

## 1. Introduction

Anti-interferon-γ (IFN-γ) autoantibody (AAb)-associated immunodeficiency (AOID) has emerged as a well-recognized disorder affecting previously healthy adults, particularly those of East Asian descent. This condition, also referred to as adult-onset immunodeficiency syndrome, presents with intracellular infections resembling those observed in acquired immunodeficiency syndrome (AIDS), yet patients consistently test negative for human immunodeficiency virus (HIV) infection [1,2]. The nature of this acquired immunodeficiency, occurring in the absence of HIV infection, underscores the critical role of IFN-γ in maintaining immune competence and highlights the need for a comprehensive understanding of the underlying immunological mechanisms.

IFN-γ is a pivotal immunoregulatory cytokine produced primarily by T cells and innate lymphoid cells. This pleiotropic cytokine exerts broad systemic effects, influencing virtually every cell type either directly or indirectly, thereby underscoring its fundamental importance in health and disease [3,4]. Deficiencies in IFN-γ signaling, whether resulting from impaired production, receptor mutations, or neutralizing autoantibodies, consistently predispose individuals to severe mycobacterial and viral infections [5,6].

The relationship between IFN-γ and autoimmune diseases reveals additional complexity in its immunomodulatory functions. While dysregulated IFN-γ expression has been linked to susceptibility to various autoimmune conditions [7], this cytokine can paradoxically either enhance or alleviate autoimmune pathology depending on the specific disease context and timing of intervention [8,9]. This dual role emphasizes the intricate balance required for optimal immune function and the potential consequences of disrupting IFN-γ signaling pathways.

The development of autoantibodies requires autoreactive B cells to overcome established tolerance mechanisms, either through failure of central tolerance or breakdown of peripheral regulatory controls. Defective B cell tolerance represents a common pathogenic feature across multiple autoantibody-mediated diseases, including autoimmune polyendocrinopathy-candidiasis-ectodermal dystrophy (APECED) [10], systemic lupus erythematosus (SLE) [11], and rheumatoid arthritis [12].

Central tolerance operates as a critical checkpoint in immune homeostasis, ensuring self-tolerance through the negative selection of developing B and T cells within primary lymphoid organs [13]. Lymphocytes expressing antigen receptors with high affinity for self-antigens undergo deletion, anergy, or receptor editing—a process that modifies antigen receptor genes to mitigate autoreactivity [14]. However, these safeguards are not absolute, and some self-reactive B and T cells escape central tolerance and migrate to peripheral tissues. Here, peripheral tolerance mechanisms attempt to maintain immune homeostasis through deletion, functional inactivation, or suppression by regulatory T (Treg) cells. Failure of these multilayered tolerance mechanisms can result in the emergence of pathogenic autoantibodies.

B cell differentiation and development are precisely orchestrated through the coordinated expression and activation of specific transcription factors and signaling molecules. The paired box domain gene 5 (PAX5) protein is critically involved in B cell progenitor development within the bone marrow and continues to influence B cell maturation and peripheral differentiation. Complementing this, B-lymphocyte-induced maturation protein 1 (BLIMP1) and X-box binding protein (XBP1) function as essential transcription factors governing antibody-secreting cell (ASC) differentiation [15,16]. The regulatory landscape is further shaped by B cell lymphoma 6 (BCL6), a transcriptional repressor that controls B-cell differentiation and facilitates germinal center formation, specialized microenvironments where B cells undergo somatic hypermutation and affinity maturation [17]. Additionally, the Kirsten Rat Sarcoma Viral Oncogene Homolog (KRAS) participates in critical signalling pathways that regulate B-cell proliferation, differentiation, and survival [18]. B cell activating factor (BAFF), a cytokine essential for B cell survival, differentiation, and activation, provides additional regulatory control over B cell homeostasis.

Understanding the genetic and molecular determinants governing autoantibody production is therefore crucial for elucidating the pathogenic mechanisms underlying AOID associated with anti-IFN-γ AAbs.

This study aimed to comprehensively characterize the immunological profiles of patients with AOID through multiple complementary approaches. Our objectives were to (1) investigate the expression patterns of genes critically involved in B cell somatic hypermutation and peripheral tolerance, including *PAX5*, *BLIMP1*, *XBP1*, *BCL6*, *KRAS*, and *BAFF*, in AOID patients compared to healthy controls; (2) comprehensively measure a panel of pro-inflammatory, anti-inflammatory, chemokine, and growth factor/regulatory cytokines in the patients’ serum samples; (3) analyze the associations between anti-IFN-γ AAbs levels, B cell-related gene expression, and cytokine profiles in serum to identify immunological signatures characteristic of AOID; and (4) investigate whether genetic variations, particularly IFN-γ nucleotide polymorphisms, contribute to self-antigen formation and autoantibody induction in these patients.

## 2. Results

### 2.1. Levels of Anti-IFN-γ AAbs in Participants

**Figure 1:**
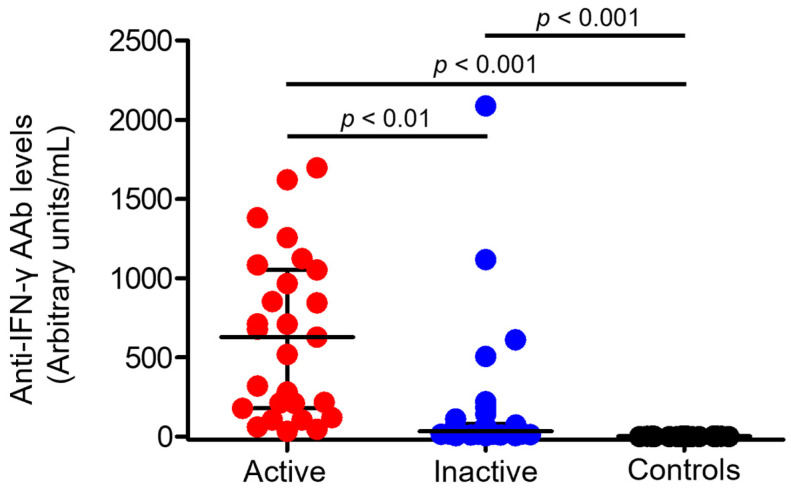
Anti-IFN-γ AAb levels in active AOID patients, inactive cases, and healthy controls. Individual anti-IFN-γ AAb levels are shown for active AOID patients (red circles), inactive cases (blue circles), and healthy controls (black circles). Horizontal lines represent median values and interquartile ranges for each group. Y-axis represents anti-IFN-γ AAb concentration in arbitrary units/mL.

Figure 1 displays the levels of AAbs against IFN-γ across the three study groups. Using a cutoff value defined as the mean antibody level of negative controls plus three standard deviations, anti-IFN-γ autoantibodies were detected in 100% of active patients, 100% of inactive cases, and 0% of healthy controls. The median anti-IFN-γ AAbs levels were 627.4 arbitrary units/mL (interquartile range [IQR] 179.1–1051) in active patients, 33.85 arbitrary units/mL (IQR 15.35–82.78) in inactive cases, and 1.54 arbitrary units/mL (IQR 0.97–2.06) in healthy controls. Anti-IFN-γ autoantibody levels in active AOID patients were significantly higher than those in the inactive group (*p* < 0.01) and healthy controls (*p* < 0.001). Similarly, inactive cases demonstrated significantly higher median anti-IFN-γ autoantibody levels compared to healthy controls (*p* < 0.001).

### 2.2. mRNA Expression Profiles of Genes Involved in Somatic Hypermutation and Peripheral Tolerance, and Their Correlation with Anti-IFN-γ AAbs

**Figure 2:**
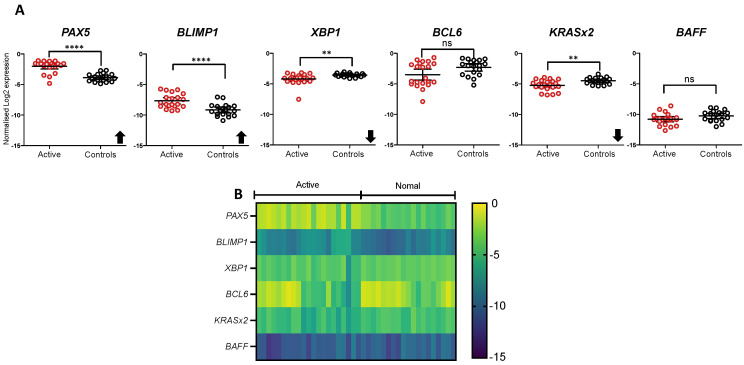
(**A**) Expression levels of *PAX5*, *BLIMP1*, *XBP1*, *BCL6*, *KRAS*, and *BAFF* mRNA in AOID patients and normal individuals. Each symbol represents the expression of genes of interest normalized against the housekeeping gene *GAPDH* (log2-fold change). Solid lines are the mean levels of mRNA expression in active AOID patients and healthy controls. **** *p* < 0.0001, and ** *p* < 0.05 compared to healthy controls; ns indicates not significant. Upward and downward arrows indicate the upregulation and downregulation of genes in active patients compared with controls, respectively. (**B**) Heat map analysis of gene expression in AOID patients and healthy controls. Gene expression levels are displayed as a color-coded heat map, with yellow indicating higher expression (0) and dark blue indicating lower expression (−15). The left panel shows expression patterns in AOID patients, while the right panel displays expression in healthy controls. Each column represents an individual participant, and each row represents a specific gene (*PAX5*, *BLIMP1*, *XBP1*, *BCL6*, *KRAS*, and *BAFF*).

**Figure 3:**
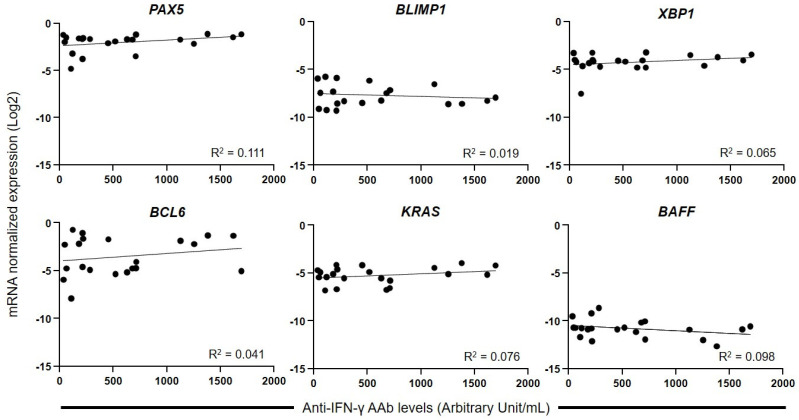
Correlation between anti-IFN-γ AAbs and gene expression levels. The levels of anti-IFN-γ AAbs in patients’ sera were measured using ELISA, and the gene expressions of *PAX5, BLIMP1*, *XBP1*, *BCL6*, *KRAS*, and *BAFF* were determined by qPCR.

To investigate mRNA expression, we examined the targeted genes of interest using qPCR and normalized the results using the housekeeping gene *GAPDH*. Due to limited cell availability in the inactive group, gene expression analysis was performed only in active AOID patients compared to healthy controls. *PAX5* and *BLIMP1* were found to be significantly more expressed in patients with AOID, with a *p*-value of less than 0.0001. In contrast, the expressions of *XBP1* and *KRAS* were statistically significantly lower in AOID patients compared to healthy controls, with *p*-values of 0.002 and 0.0048, respectively (Figure 2A and Supplementary File S1). The expressions of *BCL6* and *BAFF* did not show any statistically significant differences, with *p*-values of 0.0504 and 0.083, respectively. Heat map analysis revealed that *BAFF* and *BLIMP1* exhibited modestly low expression, while *PAX5* and *BCL6* were the two most highly expressed genes in both groups (Figure 2B). We also compared IFN-γ gene polymorphism between AOID patients and healthy controls and found that the IFN-γ nucleotide sequences within the analyzed transcript region were not different between AOID patients and controls (Supplementary File S2). No correlation was observed between the antibody levels in patients’ sera and the expression of the evaluated genes (Figure 3).

### 2.3. Comparison of Serum Cytokine, Chemokine, and Protein Levels Between AOID Patients, With or Without Current Infections, and Healthy Controls, and Their Correlation with Anti-IFN-γ AAbs Levels

**Figure 4:**
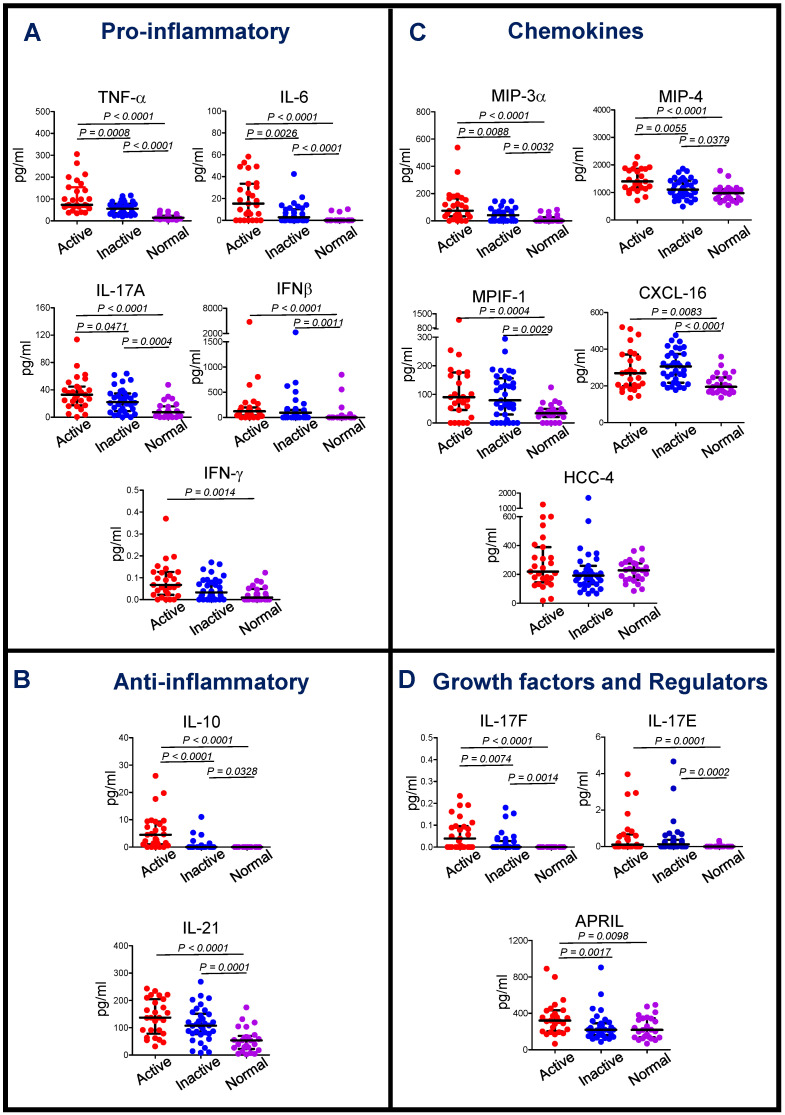
The levels of cytokine/chemokine/proteins in sera were measured using the Multiplex^®^ Assay. Individual data points show cytokine/chemokine concentrations (pg/mL) in AOID patients with active infections (red), AOID patients without current infections (blue), and healthy controls (purple). Panels show (**A**) pro-inflammatory mediators, (**B**) anti-inflammatory cytokines, (**C**) chemokines, and (**D**) growth factors/regulators. Each symbol represents one individual. The vertical lines and error bars indicate the median and interquartile range. *p* Values show statistical significance between groups.

The levels of fifteen cytokines, chemokines, and proteins in sera were determined by using the Multiplex^®^ assay. The results were divided into four functional groups of molecules, which are involved in pro-inflammatory, anti-inflammatory, chemokines, and growth factors and regulators (Figure 4 and Supplementary File S3). Patients with current infections (active group) had significantly higher levels of all pro-inflammatory cytokines than healthy controls (Figure 4A) and higher levels of TNF-α, IL-6, and IL-17A than patients with a history of infections (inactive group). Patients in the inactive group also had higher levels of pro-inflammatory cytokines than controls, except for IFN-γ. For anti-inflammatory cytokines, the levels of IL-10 in the active group were significantly higher than those in the inactive and control groups (Figure 4B). IL-10 in the inactive group was also significantly higher than healthy controls. The IL-21 levels in active and inactive groups were statistically higher than controls, whereas no differences were found between active and inactive groups. The active group also had significantly higher levels of all growth factors and regulators evaluated compared to healthy controls, and higher levels of IL-17F and APRIL when compared to the inactive group (Figure 4D). The inactive group showed significantly higher levels of IL-17E and IL-17F than controls. Similarly, we observed higher levels of all chemokines in the active group than in healthy controls, except for HCC-4 (Figure 4C). MIP-3α and MIP-4 levels in the active group were higher than in the inactive group. The levels of all evaluated chemokines in the inactive group were also higher than those of controls, except for HCC-4.

**Figure 5:**
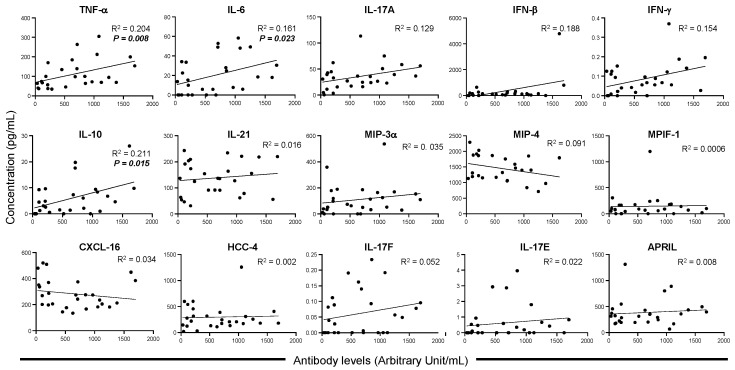
Correlation of anti-IFN-γ AAbs levels and serum inflammatory mediators in AOID patients with current infections. Scatter plots demonstrate the relationship between anti-IFN-γ AAb levels (x-axis) and cytokine/chemokine concentrations (y-axis, pg/mL) in AOID patients. Linear regression lines with R^2^ values are shown for each analyte. Each point represents an individual patient.

To investigate whether anti-IFN-γ AAbs levels were associated with serum cytokine, chemokine, and protein levels, we performed correlation analyses between antibody levels and the concentrations of 15 circulating cytokines and chemokines (Figure 5). Linear regression analysis revealed that antibody levels were positively and significantly correlated with three pro-inflammatory and immunoregulatory cytokines: TNF-α (R^2^ = 0.204, *p* = 0.008), IL-6 (R^2^ = 0.161, *p* = 0.023), and IL-10 (R^2^ = 0.211, *p* = 0.015). No significant correlations were observed between antibody levels and the remaining 12 analytes examined. Although some of these markers displayed weak positive trends (e.g., IL-17A, IL-17F, and IFN-γ), none reached statistical significance.

### 2.4. Correlation Matrix of Gene Expression and Anti-IFN-γ AAbs Levels in AOID Patients

**Figure 6:**
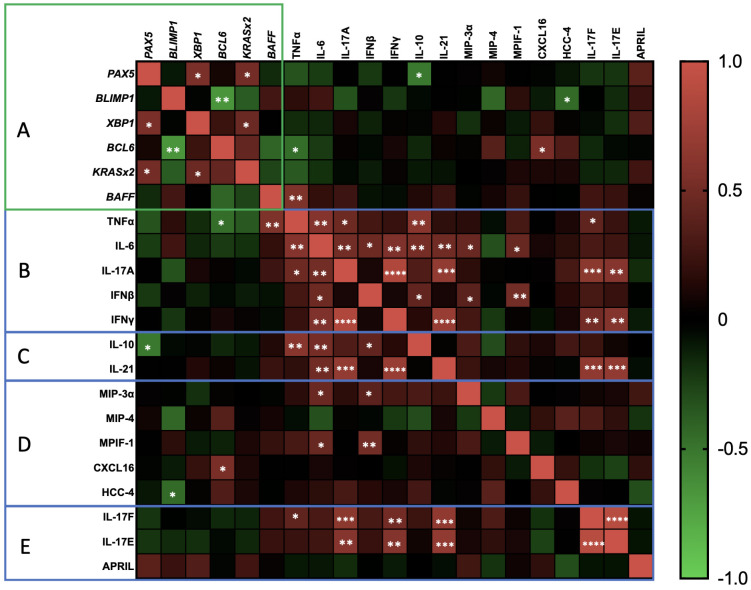
Correlation matrix of gene expression and serum inflammatory mediator levels in AOID patients. Heat map analysis displays non-parametric Spearman rank correlation between all measured parameters. Color scale ranges from −1.0 (strong negative correlation, green) to +1.0 (strong positive correlation, red), with black indicating no correlation (0). Sections are organized as follows: (**A**) Gene expression correlations among *PAX5*, *BLIMP1*, *XBP1*, *BCL6*, *KRAS*, and *BAFF*; (**B**) pro-inflammatory cytokines (TNFα, IL-6, IL-17A, IFN-β, IFN-γ); (**C**) anti-inflammatory cytokines (IL-10, IL-21); (**D**) chemokines (MIP-3α, MIP-4, MPIF-1, CXCL16, HCC-4); (**E**) growth factors and regulators (IL-17F, IL-17E, APRIL). Statistical significance is indicated by asterisks: * *p* < 0.05, ** *p* < 0.01, *** *p* < 0.001, **** *p* < 0.0001.

The correlations between the expression levels of six specific genes and the concentrations of various serum cytokines, chemokines, and regulatory proteins in AOID patients were assessed using a correlation matrix, which determines the strength and direction of relationships between two or more variables. Each cell in Figure 6 shows the correlation between two variables. Values of 1.0, −1.0, and 0 indicate positive, negative, and no correlation, respectively. We analyzed pairwise correlation among six genes (*PAX5*, *BLIMP1*, *XBP1*, *BCL6*, *KRAS*, and *BAFF*) (Figure 6A), between pro-inflammatory cytokines and anti-inflammatory cytokines, chemokines and growth factors/regulators (Figure 6B), between anti-inflammatory cytokines and the parameters described above (Figure 6C), between chemokines and the other measured variables (Figure 6D), and between growth factors/regulators and the previously mentioned parameters (Figure 6E). Out of 15 pairwise comparisons within the gene expression parameters, 4 in 15 gene–gene pairwise comparisons (26.6.%) were significant. Among these gene expression data, there was a significant positive correlation between *PAX5* and *XBP1* (*p* = 0.01), *PAX5* and *KRASx2* (*p* = 0.02), and *KRAS* and *XBP1* (*p* = 0.035). In contrast, there was a strong negative correlation between *BLIMP1* and *BCL6* (*p* = 0.001) (Figure 6A). *KRAS* and *XBP1* expressions were not correlated to any cytokines or mediators. *BAFF* and *BCL6* expression showed a positive correlation with TNF-α and CXCL16, respectively (*p* = 0.008 and 0.013). In contrast, a negative correlation was found between *BCL6* and TNF-α, *PAX5* and IL-10, and *BLIMP1* and HCC-4 (*p* = 0.038, 0.016, and 0.043, respectively). Of all inflammatory mediators examined, only CXCL16, HCC-4, and APRIL demonstrated no correlations with other cytokines or chemokines (Figure 6D,E). No statistically significant correlations were observed between gene expression and growth factors/regulators (Figure 6E).

## 3. Discussion

This study provides a comprehensive analysis of B cell-related gene expression profiles and serum cytokine levels in patients with anti-IFN-γ AAb-associated immunodeficiency, elucidating the underlying immunological dysregulation characteristic of this emerging condition. Our findings show significant alterations in B cell gene expression patterns and cytokine profiles that may contribute to AOID pathogenesis. Our findings also demonstrate a clear and significant elevation of anti-IFN-γ AAbs in both active and inactive AOID patients compared to healthy controls, with active patients showing the highest levels.

A central finding of our study is the differential expression of genes involved in B cell somatic hypermutation and peripheral tolerance in AOID patients compared to healthy controls. The significantly increased expression of *PAX5* and *BLIMP1* suggests heightened immune activity in these patients. PAX5, a transcription factor associated with the development of mature B cells prior to somatic hypermutation (SHM), normally facilitates the generation of diverse antibody repertoires through B-cell receptor (BCR) gene mutations [19]. Previous studies showed that increased *PAX5* expression enhances the proliferation and survival of B-cells [20,21], while *BLIMP1* upregulation is associated with B-cell terminal differentiation into antibody-producing plasma cells [22]. The concurrent upregulation of *PAX5* and *BLIMP1* observed in AOID patients is consistent with patterns seen in other autoimmune conditions and may reflect ongoing B cell activation processes [23,24]. This pattern mirrors observations in Sjögren’s syndrome [25], lupus [26], IgG4-related disease [27], systemic lupus erythematosus [28], and rheumatoid arthritis [29], suggesting a common pathway of aberrant B cell activation contributing to autoantibody production. However, whether these expression changes contribute to autoantibody production or represent downstream consequences of immune dysregulation cannot be determined from this cross-sectional analysis.

In contrast, the decreased expression of *XBP1*, *BCL6*, *KRAS*, and *BAFF* may reflect changes in normal B cell regulatory mechanisms. *XBP1* is involved in efficient protein folding, maturation, and degradation in the endoplasmic reticulum (ER) [30] and is critical for plasma cell differentiation into antibody-secreting cells [31]. *KRAS* is essential for early B cell development and late B cell maturation [18,32]. Downregulation of *XBP1* and *KRAS* expression might contribute to unresolved ER stress and impairment of late B cell maturation, respectively, possibly contributing to developmental abnormalities. These findings align with observations in systemic sclerosis [33] and pediatric Rosai-Dorfman syndrome and systemic lupus erythematosus [34].

*BCL6* downregulation may be of particular interest, as this transcriptional repressor is involved in maintaining germinal center B cells and preventing premature differentiation [17]. Reduced *BCL6* expression might contribute to changes in germinal center formation and could influence B cell affinity maturation and class switching [35], possibly resulting in autoantibodies with reduced binding affinity or broader antigen recognition. Direct evidence of downregulation of *BCL6* as a primary pathogenic mechanism for human autoimmune diseases is less common compared to its upregulation. However, it has been shown in an animal model that the deficiency of *BCL6* affects macrophage polarization, leading to an exacerbated autoimmune encephalomyelitis [36]. Heat map analysis revealed *PAX5* and *BCL6* among the most highly expressed genes evaluated, suggesting a complex regulatory landscape where enhanced differentiation pathways (*PAX5*) may coexist with potentially limited regulatory control (*BCL6*), which may reflect changes in B-cell differentiation.

Our investigation of IFN-γ nucleotide sequences within the analyzed transcript region revealed no significant differences between AOID patients and healthy controls. This finding suggests that genetic polymorphism in the IFN-γ gene alone may not fully account for anti-IFN-γ AAb generation or AOID predisposition.

The comprehensive analysis of serum cytokines, chemokines, and proteins revealed profound immune dysregulation in AOID patients. Those with current infections (“active” group) exhibited significantly elevated levels of pro-inflammatory cytokines (TNF-α, IL-6, IL-17A), anti-inflammatory cytokines (IL-10, IL-21), growth factors/regulators (IL-17F, APRIL), and chemokines (MIP-3α, MIP-4, CXCL16, MPIF-1) compared to healthy controls. This widespread elevation indicates chronic immune activation and inflammation. Even patients without active infections (“inactive” group) demonstrated elevated cytokine levels compared to healthy controls, suggesting persistent low-grade inflammation and ongoing regulatory attempts to control immune responses. The elevation of anti-inflammatory cytokine, IL-10, represents a compensatory mechanism to inflammation. However, its apparent inability to suppress autoantibody production could suggest some impairment in regulatory function.

Recent research has illuminated the complex interplay between cytokines and gene expression in immune regulation and autoimmune disease development. Pro-inflammatory cytokines can downregulate *XBP1*, *BCL6*, *KRAS*, and *BAFF* expression across various cell types. TNF-α inhibits *XBP1* expression in B cells, leading to impaired immunoglobulin secretion and B-cell differentiation [37]. IL-6 downregulates *KRAS* expression in human lung cancer cells, while IL-21 and IL-6 suppress *BCL6* in Follicular helper T cells [38]. *BAFF* expression can also be inhibited by IFN-γ and TNF-α, affecting B-cell survival and differentiation [39,40]. Cytokine IL-17 may downregulate *XBP1* under specific conditions, potentially contributing to the development of autoimmune disease [41]. The connection between *XBP1* and B cell function is particularly relevant [31,42], as blocking the *XBP1* pathway can inhibit B cell hyperactivity and plasma cell overproduction in mouse models, suggesting therapeutic potential for conditions such as lupus nephritis. Notably, the low *XBP1* expression observed in our AOID patients parallels findings in SLE, where *XBP1* is repressed by BLIMP1 [24]. Correlation matrix analysis revealed significant regulatory relationships between gene expression and cytokine levels. Positive correlations between *KRAS* and *PAX5*, *KRAS* and *XBP1*, and *PAX5* and *XBP1* may suggest coordinated regulatory networks involved in modulating immune responses. The negative correlation between *BLIMP1* and *BCL6* could further support the concept of balanced regulatory mechanisms that may be altered in AOID.

The lack of correlation between anti-IFN-γ AAb levels and gene expression suggests that while these genes may play a role in immune response modulation, their expression appears not to be directly associated with antibody quantity. This finding could indicate that alternative regulatory mechanisms or additional factors may influence antibody production in AOID patients, suggesting the need for further investigation into complex pathways potentially involved in autoantibody generation.

Our study had some limitations worth noting. The relatively small sample size and the exclusion of inactive AOID patients from gene expression analysis due to sample constraints limited our ability to compare gene expression profiles across different disease states. As cell availability from the inactive group was limited, qPCR analysis was restricted to active AOID patients and healthy controls. Consequently, we cannot rule out that some altered gene expression profiles could be driven by ongoing opportunistic infections/systemic inflammation rather than primary autoantibody pathobiology. Future studies comparing active and inactive disease states are necessary. Inclusion of patients with comparable opportunistic infections but without anti-IFN-γ autoantibodies would also strengthen the study. Unfortunately, enrolling such a cohort was beyond the scope and sample availability of the current cross-sectional study. Given the exploratory nature of this study across multiple cytokine and gene-expression analyses, unadjusted *p*-values are reported. Consequently, these findings should be interpreted as hypothesis-generating and warrant validation in larger, independent cohorts with formal multiple-testing adjustments. Although our findings demonstrate distinct antibody and cytokine patterns, we did not perform functional neutralization assays to directly assess biological activity. This represents an important limitation of the current study, and functional assays will be necessary in follow-up studies to confirm the mechanistic significance of these responses. Additionally, we focused primarily on B cells; investigation of non-B cell populations may provide further insight, as other cell types could express the genes of interest. The cross-sectional design also prevented the establishment of causal relationships between gene expression changes and disease progression. Longitudinal studies would be valuable to better understand the temporal dynamics of these changes.

## 4. Materials and Methods

### 4.1. Study Population

We enrolled 63 patients in this cross-sectional study at Chiang Mai University Hospital, Chiang Mai, Thailand. These patients met specific criteria: they were having (active group) or had a history of (inactive group) culture or histopathology-proven opportunistic infections, tested positive for anti-IFN-γ AAbs, and were constantly confirmed HIV-negative by enzyme-linked immunosorbent assay. The infections included disseminated non-tuberculous mycobacterial infection, Salmonellosis, Candidiasis, Herpes zoster virus infection, talaromycosis, cryptococcosis, and Histoplasmosis. Participants comprised 32 males and 31 females, with ages ranging from 39–77 years and 41–71 years, respectively. Thirty healthy controls (20 females and 10 males, aged 39–61 years) were recruited based on the absence of recent infections (within 30 days of enrolment) and no prior history of Mycobacterium tuberculosis infection. None of the participants were receiving immunosuppressive drugs. The study was approved by the ethics committees of the Faculty of Medicine (105/2557) and the Research Institute for Health Sciences (13/56), Chiang Mai University. All procedures performed in studies involving human participants were in accordance with the ethical standards and with the 1964 Helsinki Declaration. Written informed consent was obtained from all individual participants before they were enrolled in the study.

### 4.2. Blood Collection

Venous blood was collected, and peripheral blood mononuclear cells (PBMC) were isolated by gradient centrifugation as described previously [43]. Serum samples were collected and stored at −70 °C for measurement of anti-IFN-γ AAbs levels and cytokine levels by Multiplex bead assay (Milliplex^®^, Merck, Darmstadt, Germany).

### 4.3. B Cell Isolation

B cells were isolated from PBMCs through negative selection using the MACS B cell isolation kit II (Miltenyi Biotec GmbH, Bergish Gladbach, Germany) according to the manufacturer’s protocols. Briefly, PBMCs from 21 active patients and 19 healthy controls were incubated with a biotin-conjugated Ab cocktail, followed by incubation with anti-biotin microbeads and the unbound B cells were separated using the MS MACS^®^ separation column and MiniMACS™ separator (Miltenyi Biotec GmbH, Bergish Gladbach, Germany). Isolation efficiency was evaluated by staining with CD19 and CD3 and analyzed using a flow cytometer (Cyan ADP analyser, Beckman Coulter, Brea, CA, USA). The average B-cell isolation purity was 87.2 ± 10.32 as measured by CD3^−^ CD19^+^ flow cytometry. Isolated B cells were preserved with the RNA-later (Thermo Fisher Scientific, Waltham, MA, USA) and stored at −70 °C before RNA extraction.

### 4.4. RNA Extraction and cDNA Synthesis

The total RNA was extracted from isolated B cells using the Nucleospin^®^ RNA kit (Macherey-Nagel, Düren, Germany), and the extracted material was pre-treated with DNase according to the manufacturer’s protocols. Reverse transcription was then performed using the ReverTraAce^®^ qPCR RT Master Mix with gDNA Remover (FSQ-301, TOYOBO, Osaka, Japan) following the determination of RNA concentrations using a Nanodrop spectrometer (NanoDrop™ 2000, Thermo Scientific, Wilmington, DE, USA). cDNA synthesis was carried out following the instructions of ReverTra Ace™ qPCR RT Master Mix with gDNA Remover (TOYOBO, Japan).

### 4.5. Real-Time PCR (qPCR) Analysis

cDNA was prepared from isolated B cells as described above. The qPCR and melt curve analysis were performed by using 7 primers, namely, *GAPDH*, *BAFF*, *XBP-1*, *KRAS*, *BLIMP-1, PAX-5*, and *BCL-6*, which were designed using 20 NCBI/Primer-BLAST (Primer3 and BLAST) (National Center for Biotechnology Information, Bethesda, MD, USA; https://www.ncbi.nlm.nih.gov/tools/primer-blast/; accessed on 15 February 2018) and commercially synthesized by Bio Basic Inc. (Markham, ON, Canada). The sequences of the primers are listed in Table 1. The qPCR and melt curve analysis were performed following the instructions of the THUNDERBIRD^®^ SYBR^®^ qPCR Mix kit (QPS-201, TOYOBO, Osaka, Japan). Briefly, a total of 20 µL reaction comprised 0.2–0.4 mM of each forward and reverse primer, 0.1x ROX dye, Thunderbird PCR mix, and 1 µL of cDNA template. The qPCR reactions were conducted using the ABI 7500 Fast Real-Time PCR system (Applied Biosystems, Thermo Scientific, Wilmington, DE, USA). The amplification conditions were as follows: an initial incubation at 95 °C for 1 min, followed by 50 cycles of 95 °C for 15 s and 64 °C for 30 s. After that, the melt curve was done at the final stage of the reaction. Gene-specific in-house DNA standards were prepared as 10-fold serial dilutions ranging from 0.1 to 10,000 fg/µL and were included in each assay to generate standard curves for quantity estimation. Each target and reference-gene assay was performed in duplicate across two separate qPCR runs to generate four data sets. Ct values from individual wells were independently converted to quantities using the corresponding gene-specific standard curve. Relative gene expression was calculated using normalized log_2 transformed values or the 2^−ΔΔC^_T_ method relative to *GAPDH*. *GAPDH* stability was verified prior to normalization.

### 4.6. IFN-γ Nucleotide Sequencing

PBMC were stimulated with medium alone or with 50 ng/mL phorbol myristate acetate (PMA) and 500 ng/mL ionomycin for 24 h in the presence of brefeldin A and Monensin. The target region included 530 base pairs of the 105 to 610 upstream sequences of human IFN-γ mRNA, which were amplified by reverse transcriptase PCR using the primers: IFN-γ-F: 5′-GGCTTAATTCTCTCGGAAACGATG-3′ and IFN-γ-R: 5′-GACAACCATTACTGGGATGCTCTTC-3′. Total PCR reaction was performed in a 25 µL reaction vessel consisting of 1x KOD plus buffer, 1.5 mM MgSO4, 0.2 mM dNTPs, 0.15 mM each of forward and reverse primers, and 1 U of KOD Plus NEO DNA polymerase (KOD-401, TOYOBO, Japan). The PCR reaction started with a 2 min pre-denaturation step at 94 °C, followed by 35 cycles consisting of 10 s at 98 °C, 30 s at 55 °C, and 30 s at 68 °C, with a 7 min extension step at 68 °C. PCR products were analyzed on a 2% agarose gel using an electrophoresis voltage of 100 volts for 45 min. The results were visualised under UV light after staining with ethidium bromide. PCR products were then prepared and sent to Bio Basic Inc. (Markham, ON, Canada), where the purification and sequencing services were performed.

### 4.7. Determination of Cytokines by Multiplex^®^ Assay

Multiplex^®^ MAP human Th17 magnetic bead panel and human cytokine/chemokine magnetic bead panel IV kits were purchased from Merck Millipore (EMD Millipore Corporation, Burlington, MA, USA). Human Th17 panel consists of fluorescent-coded magnetic beads coated with specific antibodies against tumor necrosis factor (TNF)-α, IFN-γ, interleukin (IL)-6, IL-10, IL-17A, IL-17E/IL-25, IL-17F, IL-21, and MIP-3α/CCL20. The Human Cytokine/Chemokine Magnetic Bead Panel IV kit (EMD Millipore Corporation, Burlington, MA, USA) was used for the quantitation of APRIL, IFN-γ, CXCL16, HCC-4, MIP-4/PARC, and MPIF-1. The assay was performed following the manufacturer’s instructions. Briefly, neat sera (for Human Th17 kit: (EMD Millipore Corporation, Burlington, MA, USA)) or 1:4 diluted sera (for Human cytokine/chemokine kit (EMD Millipore Corporation, Burlington, MA, USA)) were analyzed in duplicates. Twenty-five microliters of samples or standards were added to bead-containing wells of the assay microplate. Multiplex standards were generated according to the assay kit and added to the appropriate wells. After overnight incubation at 4 °C, the plates were washed according to the provided instructions, and 25 μL of detection antibodies were added. The reaction was allowed to incubate for 1 h at room temperature. After this incubation, 25 μL of streptavidin-phycoerythrin was added, and the plate was agitated on a plate shaker for 30 min at room temperature.

### 4.8. Determination of Anti-IFN-γ Autoantibody by ELISA

The levels of anti-IFN-γ AAbs were assessed by indirect enzyme-linked immunosorbent assay (ELISA), as previously described [43]. Sera were diluted to a working dilution of 1:1000, and all assays were performed in duplicate for each sample, with an intra-assay coefficient of variation (CV) of less than 5%. The cutoff value was defined as the mean value of healthy controls plus 3 standard deviations.

### 4.9. Statistical Analysis

Student’s *t*-test was used to compare the mean of gene expression values between two groups. The heat map of gene expression was analyzed using a log2 scale. Spearman’s correlation coefficient was used to test for correlations between the cytokines and genes. The Mann–Whitney U test was used to analyze the differences in the cytokine levels between groups. All the analyses were performed using the GraphPad Prism Software Version 5.00 (Boston, MA, USA).

## 5. Conclusions

In summary, this study identifies notable changes in B cell-related gene expression and a broad inflammatory cytokine profile in patients with AOID. The altered expression of *PAX5*, *BLIMP1*, *XBP1*, *BCL6*, *KRAS*, and *BAFF* may reflect perturbations in B cell differentiation and tolerance mechanisms, potentially contributing to the autoimmune phenotype. Elevated levels of pro-inflammatory and anti-inflammatory cytokines, chemokines, and growth factors suggest a state of chronic immune activation, with certain cytokines showing associations with anti-IFN-γ autoantibody levels. These findings may enhance our understanding of this rare but serious condition and could potentially inform the development of targeted therapeutic approaches. Larger cohort studies are warranted to validate these findings and to further explore the therapeutic implications of modulating the identified pathways.

## Figures and Tables

**Table 1:** Primer sequences for Real-time PCR.

Gene	Forward Sequence	Reverse Sequence
*GAPDH*	GGTGTGAACCATGAGAAGTATGA	GAGTCCTTCCACGATACCAAAG
*PAX5*	TACTCCATCAGCGGCATCCT	CTCCTGAATACCTTCGTCTCTCTTG
*BLIMP1*	CAGGTCTGCCACAAGAGATTTA	CACCTTGCATTGGTATGGTTTC
*XBP1*	AGGAGTTAAGACAGCGCTTGG	AGAGGTGCACGTAGTCTGAGTGCTG
*BCL6*	CTGCAGATGGAGCATGTTGT	TCTTCACGAGGAGGCTTGAT
*KRAS*	GACTCTGAAGATGTACTATGGTCCTA	CATCATCAACACCCTGTCTTGTC
*BAFF*	CGTTCAGGGTCCAGAAGAAA	CTGAGAAGCCATGGAACAAATG

## Data Availability

The original contributions presented in this study are included in the article/Supplementary Materials. Further inquiries can be directed to the corresponding author.

## Supplementary Materials

The following supporting information can be downloaded at: https://www.mdpi.com/article/10.3390/ijms27156998/s1.

## Abbreviations

The following abbreviations are used in this manuscript:

AAb	Autoantibody
AOID	Adult-onset immunodeficiency
IFN	Interferon
AIDS	Acquired immunodeficiency syndrome
HIV	Human immunodeficiency virus
APECED	Autoimmune polyendocrinopathy-candidiasis-ectodermal dystrophy
SLE	Systemic lupus erythematosus
Treg	Regulatory T cells
PAX5	Paired box domain gene 5
BLIMP1	B-lymphocyte-induced maturation protein 1
XBP1	X-box binding protein
ASC	Antibody-secreting cell
BCL6	B cell lymphoma 6
KRAS	Kirsten Rat Sarcoma Viral Oncogene Homolog
BAFF	B cell activating factor
PBMC	Peripheral blood mononuclear cells
PCR	Polymerase chain reaction
TNF-α	Tumor necrosis factor-α
IL	Interleukin
ELISA	Enzyme-linked immunosorbent assay
IQR	Interquartile range
GAPDH	Glyceraldehyde 3-phosphate dehydrogenase
APRIL	A proliferation-inducing ligand
SHM	Somatic hypermutation
BCR	B-cell receptor
