# Supplementary material for: Investigation of Cytokine Profiles, the Expression of Genes Involved in Somatic Hypermutation and Peripheral Tolerance, and Their Correlation with Autoantibody Levels in Northern-Thai Immunodeficient Patients Carrying Anti-Interferon-γ Autoantibodies
Source: Int J Mol Sci. 2026 Aug 4;27(15):6998. doi: 10.3390/ijms27156998 (PMC13466844; doi:10.3390/ijms27156998)
Supplement: Supplementary file 1 [file ijms-27-06998-s001.zip › Supplementary File S2 IFNg nucleotide sequences.pdf]

Supplementary File S2. Interferon-γ nucleotide sequences from AOID patients (A; n = 25) and healthy controls (N; n = 18). Sequencing analysis was performed in three independent sets.

SET 1

```
N005        -------------------------------------------GGAACATTTCTTGGCAT      17
N003        ------------------------------------TTTCCCAGGCTTATATCTT-----      19
A005        --------------------------------------AAAAGGTTTAATCCTTT-----      17
A006        ------------------------------------CTACCAAGTTTAAATCCTT-----      19
A012        -------------------------------------AACCAAGCTTATATCCTT-----      18
A001        ---------------------------------------AACCAGCTAT-ATCTT-----      15
N013        -------------------------------------AAAAAAGGTTATAATCTT-----      18
N011        ---------------------------------------AAACAGCTAT-ATCTT-----      15
N004        --------------------------------------AAAAAGCTTAT-ATCTT-----      16
N008        --------------------------------------AACAAGCTTAT-ATCTT-----      16
N014        -------------------------------------ATTCAAGGTTTATATCTT----T      19
A013        --------------------------------------AAAAAGCTTAATATC-T----T      17
A023        -------------------------------------AACAAAGGCTTATATC-T----T      18
A007        --------------------------------------AACAAAGCTTATATC-T----T      17
A020        ---------------------------------------AAAAAGCTTATATC-T----T      16
A003        --------------------------------------ATCCAAGGTTATATCCT----T      18
A010        ---------------------------------------ATCAAGGTTATATC-T----T      16
N012        -------------------------------------ATCCCAAGTTTATATC-T----T      18
N005R       GAAATTCTTTTGGCTTAATTCTCTCGGAAACGATGAAATATACAAGTTATATCTT-----      55
A012R       --------TGGGCTTAAATTTTCTCGGAAACGATGAAATATACAAGTTATATCTT-----      47
N012R       -----TCATGGGCTTTATTTTTCTCGGAAACGATGAAATATACAAGTTATATCTT-----      50
A020R       -----TCCTTGGCTTAATTTTTCTCGGAAACGATGAAATATACAAGTTATATCTT-----      50
N014R       ------TCTTGGCTTTATTTTTCTCGGAAACGATGAAATATACAAGTTATATCTT-----      49
N011R       ------CACAGGGCCTTATTTTCTCGGAAACGATGAAATATACAAGTTATATCTT-----      49
N003R       -----TTTCTGGGTTTTATTTTTTTGGAAACGATGAAATATACAAGTTATATCTT-----      50
A013R       -----TGTATGGGCTTAATTCTCTCGGAAACGATGAAATATACAAGTTATATCTT-----      50
A003R       ---------TGGGCTTAATTTTCTCGGAAACGATGAAATATACAAGTTATATCTT-----      46
A005R       -------TATTGGCCTAATTTTCTCGGAAACGATGAAATATACAAGTTATATCTT-----      48
A023R       -------TATGGGCTTAATTTTCTCGGAAACGATGAAATATACAAGTTATATCTT-----      48
N013R       -----TGTATGGGCTTAATTTTCTCGGAAACGATGAAATATACAAGTTATATCTT-----      50
N004R       ----TTAGTGGGTTTTATTTTTCTCGGAAACGATGAAATATACAAGTTATATCTT-----      51
A010R       --------TGGGCTTAATTTTTCTCGGAAACGATGAAATATACAAGTTATATCTT-----      47
N008R       ----TTCTTTTGGTTTATTTTTCTCGGAAACGATGAAATATACAAGTTATATCTT-----      51
A006R       -----TCTTTGGCTTAATTTTCCTCGGAAACGATGAAATATACAAGTTATATCTT-----      50
A001R       -----------------------TCGGAAACGATGAAATATACAAGTTATATCTT-----      32
A007R       -----TATTGGCCTTAATTTTTCTCGGAAACGATGAAATATACAAGTTATATCTT-----      50
                                                                  *

N005        GAGCTTCCCAGCTCGGCATCGATTTGGGTTCTCTTGGCTGTTACTGCCAGGACCCATATG      77
N003        G-GCTTTTCAGCTCTGCATCGTTTTGGGTTCTCTTGGCTGTTACTGCCAGGACCCATATG      78
A005        G-GCTTTTCAGCTCTGCATCGTTTTGGGTTCTCTTGGCTGTTACTGCCAGGACCCATATG      76
A006        G-GCTTTTCAGCTCTGCATCGTTTTGGGTTCTCTTGGCTGTTACTGCCAGGACCCATATG      78
A012        G-GCTTTTCAGCTCTGCATCGTTTTGGGTTCTCTTGGCTGTTACTGCCAGGACCCATATG      77
A001        G-GCTTTTCAGCTCTGCATCGTTTTGGGTTCTCTTGGCTGTTACTGCCAGGACCCATATG      74
N013        G-GCTTTTCAGCTCTGCATCGTTTTGGGTTCTCTTGGCTGTTACTGCCAGGACCCATATG      77
N011        G-GCTTTTCAGCTCTGCATCGTTTTGGGTTCTCTTGGCTGTTACTGCCAGGACCCATATG      74
N004        G-GCTTTTCAGCTCTGCATCGTTTTGGGTTCTCTTGGCTGTTACTGCCAGGACCCATATG      75
N008        G-GCTTTTCAGCTCTGCATCGTTTTGGGTTCTCTTGGCTGTTACTGCCAGGACCCATATG      75
N014        GGCCTTTTCAGCTCTGCATCGTTTTGGGTTCTCTTGGCTGTTACTGCCAGGACCCATATG      79
A013        GGC-TTTTCAGCTCTGCATCGTTTTGGGTTCTCTTGGCTGTTACTGCCAGGACCCATATG      76
A023        GGC-TTTTCAGCTCTGCATCGTTTTGGGTTCTCTTGGCTGTTACTGCCAGGACCCATATG      77
A007        GGC-TTTTCAGCTCTGCATCGTTTTGGGTTCTCTTGGCTGTTACTGCCAGGACCCATATG      76
A020        GGC-TTTTCAGCTCTGCATCGTTTTGGGTTCTCTTGGCTGTTACTGCCAGGACCCATATG      75
A003        GGC-TTTTCAGCTCTGCATCGTTTTGGGTTCTCTTGGCTGTTACTGCCAGGACCCATATG      77
A010        GGC-TTTTCAGCTCTGCATCGTTTTGGGTTCTCTTGGCTGTTACTGCCAGGACCCATATG      75
N012        GGC-TTTTCAGCTCTGCATCGTTTTGGGTTCTCTTGGCTGTTACTGCCAGGACCCATATG      77
N005R       G-GCTTTTCAGCTCTGCATCGTTTTGGGTTCTCTTGGCTGTTACTGCCAGGACCCATATG      114
```

```
A012R      G-GCTTTTCAGCTCTGCATCGTTTTGGGTTCTCTTGGCTGTTACTGCCAGGACCCATATG     106
N012R      G-GCTTTTCAGCTCTGCATCGTTTTGGGTTCTCTTGGCTGTTACTGCCAGGACCCATATG     109
A020R      G-GCTTTTCAGCTCTGCATCGTTTTGGGTTCTCTTGGCTGTTACTGCCAGGACCCATATG     109
N014R      G-GCTTTTCAGCTCTGCATCGTTTTGGGTTCTCTTGGCTGTTACTGCCAGGACCCATATG     108
N011R      G-GCTTTTCAGCTCTGCATCGTTTTGGGTTCTCTTGGCTGTTACTGCCAGGACCCATATG     108
N003R      G-GCTTTTCAGCTCTGCATCGTTTTGGGTTCTCTTGGCTGTTACTGCCAGGACCCATATG     109
A013R      G-GCTTTTCAGCTCTGCATCGTTTTGGGTTCTCTTGGCTGTTACTGCCAGGACCCATATG     109
A003R      G-GCTTTTCAGCTCTGCATCGTTTTGGGTTCTCTTGGCTGTTACTGCCAGGACCCATATG     105
A005R      G-GCTTTTCAGCTCTGCATCGTTTTGGGTTCTCTTGGCTGTTACTGCCAGGACCCATATG     107
A023R      G-GCTTTTCAGCTCTGCATCGTTTTGGGTTCTCTTGGCTGTTACTGCCAGGACCCATATG     107
N013R      G-GCTTTTCAGCTCTGCATCGTTTTGGGTTCTCTTGGCTGTTACTGCCAGGACCCATATG     109
N004R      G-GCTTTTCAGCTCTGCATCGTTTTGGGTTCTCTTGGCTGTTACTGCCAGGACCCATATG     110
A010R      G-GCTTTTCAGCTCTGCATCGTTTTGGGTTCTCTTGGCTGTTACTGCCAGGACCCATATG     106
N008R      G-GCTTTTCAGCTCTGCATCGTTTTGGGTTCTCTTGGCTGTTACTGCCAGGACCCATATG     110
A006R      G-GCTTTTCAGCTCTGCATCGTTTTGGGTTCTCTTGGCTGTTACTGCCAGGACCCATATG     109
A001R      G-GCTTTTCAGCTCTGCATCGTTTTGGGTTCTCTTGGCTGTTACTGCCAGGACCCATATG     91
A007R      G-GCTTTTCAGCTCTGCATCGTTTTGGGTTCTCTTGGCTGTTACTGCCAGGACCCATATG     109
           *   **  ****** ****** **************************************

N005       TAAAAGAAGCAGAAAACCTTAAGAAATATTTTAATGCAGGTCATTCAGATGTAGCGGATA     137
N003       TAAAAGAAGCAGAAAACCTTAAGAAATATTTTAATGCAGGTCATTCAGATGTAGCGGATA     138
A005       TAAAAGAAGCAGAAAACCTTAAGAAATATTTTAATGCAGGTCATTCAGATGTAGCGGATA     136
A006       TAAAAGAAGCAGAAAACCTTAAGAAATATTTTAATGCAGGTCATTCAGATGTAGCGGATA     138
A012       TAAAAGAAGCAGAAAACCTTAAGAAATATTTTAATGCAGGTCATTCAGATGTAGCGGATA     137
A001       TAAAAGAAGCAGAAAACCTTAAGAAATATTTTAATGCAGGTCATTCAGATGTAGCGGATA     134
N013       TAAAAGAAGCAGAAAACCTTAAGAAATATTTTAATGCAGGTCATTCAGATGTAGCGGATA     137
N011       TAAAAGAAGCAGAAAACCTTAAGAAATATTTTAATGCAGGTCATTCAGATGTAGCGGATA     134
N004       TAAAAGAAGCAGAAAACCTTAAGAAATATTTTAATGCAGGTCATTCAGATGTAGCGGATA     135
N008       TAAAAGAAGCAGAAAACCTTAAGAAATATTTTAATGCAGGTCATTCAGATGTAGCGGATA     135
N014       TAAAAGAAGCAGAAAACCTTAAGAAATATTTTAATGCAGGTCATTCAGATGTAGCGGATA     139
A013       TAAAAGAAGCAGAAAACCTTAAGAAATATTTTAATGCAGGTCATTCAGATGTAGCGGATA     136
A023       TAAAAGAAGCAGAAAACCTTAAGAAATATTTTAATGCAGGTCATTCAGATGTAGCGGATA     137
A007       TAAAAGAAGCAGAAAACCTTAAGAAATATTTTAATGCAGGTCATTCAGATGTAGCGGATA     136
A020       TAAAAGAAGCAGAAAACCTTAAGAAATATTTTAATGCAGGTCATTCAGATGTAGCGGATA     135
A003       TAAAAGAAGCAGAAAACCTTAAGAAATATTTTAATGCAGGTCATTCAGATGTAGCGGATA     137
A010       TAAAAGAAGCAGAAAACCTTAAGAAATATTTTAATGCAGGTCATTCAGATGTAGCGGATA     135
N012       TAAAAGAAGCAGAAAACCTTAAGAAATATTTTAATGCAGGTCATTCAGATGTAGCGGATA     137
N005R      TAAAAGAAGCAGAAAACCTTAAGAAATATTTTAATGCAGGTCATTCAGATGTAGCGGATA     174
A012R      TAAAAGAAGCAGAAAACCTTAAGAAATATTTTAATGCAGGTCATTCAGATGTAGCGGATA     166
N012R      TAAAAGAAGCAGAAAACCTTAAGAAATATTTTAATGCAGGTCATTCAGATGTAGCGGATA     169
A020R      TAAAAGAAGCAGAAAACCTTAAGAAATATTTTAATGCAGGTCATTCAGATGTAGCGGATA     169
N014R      TAAAAGAAGCAGAAAACCTTAAGAAATATTTTAATGCAGGTCATTCAGATGTAGCGGATA     168
N011R      TAAAAGAAGCAGAAAACCTTAAGAAATATTTTAATGCAGGTCATTCAGATGTAGCGGATA     168
N003R      TAAAAGAAGCAGAAAACCTTAAGAAATATTTTAATGCAGGTCATTCAGATGTAGCGGATA     169
A013R      TAAAAGAAGCAGAAAACCTTAAGAAATATTTTAATGCAGGTCATTCAGATGTAGCGGATA     169
A003R      TAAAAGAAGCAGAAAACCTTAAGAAATATTTTAATGCAGGTCATTCAGATGTAGCGGATA     165
A005R      TAAAAGAAGCAGAAAACCTTAAGAAATATTTTAATGCAGGTCATTCAGATGTAGCGGATA     167
A023R      TAAAAGAAGCAGAAAACCTTAAGAAATATTTTAATGCAGGTCATTCAGATGTAGCGGATA     167
N013R      TAAAAGAAGCAGAAAACCTTAAGAAATATTTTAATGCAGGTCATTCAGATGTAGCGGATA     169
N004R      TAAAAGAAGCAGAAAACCTTAAGAAATATTTTAATGCAGGTCATTCAGATGTAGCGGATA     170
A010R      TAAAAGAAGCAGAAAACCTTAAGAAATATTTTAATGCAGGTCATTCAGATGTAGCGGATA     166
N008R      TAAAAGAAGCAGAAAACCTTAAGAAATATTTTAATGCAGGTCATTCAGATGTAGCGGATA     170
A006R      TAAAAGAAGCAGAAAACCTTAAGAAATATTTTAATGCAGGTCATTCAGATGTAGCGGATA     169
A001R      TAAAAGAAGCAGAAAACCTTAAGAAATATTTTAATGCAGGTCATTCAGATGTAGCGGATA     151
A007R      TAAAAGAAGCAGAAAACCTTAAGAAATATTTTAATGCAGGTCATTCAGATGTAGCGGATA     169
           ************************************************************

N005       ATGGAACTCTTTTCTTAGGCATTTTGAAGAATTGGAAAGAGGAGAGTGACAGAAAAATAA     197
N003       ATGGAACTCTTTTCTTAGGCATTTTGAAGAATTGGAAAGAGGAGAGTGACAGAAAAATAA     198
A005       ATGGAACTCTTTTCTTAGGCATTTTGAAGAATTGGAAAGAGGAGAGTGACAGAAAAATAA     196
A006       ATGGAACTCTTTTCTTAGGCATTTTGAAGAATTGGAAAGAGGAGAGTGACAGAAAAATAA     198
A012       ATGGAACTCTTTTCTTAGGCATTTTGAAGAATTGGAAAGAGGAGAGTGACAGAAAAATAA     197
A001       ATGGAACTCTTTTCTTAGGCATTTTGAAGAATTGGAAAGAGGAGAGTGACAGAAAAATAA     194
```

```
N013      ATGGAACTCTTTTCTTAGGCATTTTGAAGAATTGGAAAGAGGAGAGTGACAGAAAAATAA   197
N011      ATGGAACTCTTTTCTTAGGCATTTTGAAGAATTGGAAAGAGGAGAGTGACAGAAAAATAA   194
N004      ATGGAACTCTTTTCTTAGGCATTTTGAAGAATTGGAAAGAGGAGAGTGACAGAAAAATAA   195
N008      ATGGAACTCTTTTCTTAGGCATTTTGAAGAATTGGAAAGAGGAGAGTGACAGAAAAATAA   195
N014      ATGGAACTCTTTTCTTAGGCATTTTGAAGAATTGGAAAGAGGAGAGTGACAGAAAAATAA   199
A013      ATGGAACTCTTTTCTTAGGCATTTTGAAGAATTGGAAAGAGGAGAGTGACAGAAAAATAA   196
A023      ATGGAACTCTTTTCTTAGGCATTTTGAAGAATTGGAAAGAGGAGAGTGACAGAAAAATAA   197
A007      ATGGAACTCTTTTCTTAGGCATTTTGAAGAATTGGAAAGAGGAGAGTGACAGAAAAATAA   196
A020      ATGGAACTCTTTTCTTAGGCATTTTGAAGAATTGGAAAGAGGAGAGTGACAGAAAAATAA   195
A003      ATGGAACTCTTTTCTTAGGCATTTTGAAGAATTGGAAAGAGGAGAGTGACAGAAAAATAA   197
A010      ATGGAACTCTTTTCTTAGGCATTTTGAAGAATTGGAAAGAGGAGAGTGACAGAAAAATAA   195
N012      ATGGAACTCTTTTCTTAGGCATTTTGAAGAATTGGAAAGAGGAGAGTGACAGAAAAATAA   197
N005R     ATGGAACTCTTTTCTTAGGCATTTTGAAGAATTGGAAAGAGGAGAGTGACAGAAAAATAA   234
A012R     ATGGAACTCTTTTCTTAGGCATTTTGAAGAATTGGAAAGAGGAGAGTGACAGAAAAATAA   226
N012R     ATGGAACTCTTTTCTTAGGCATTTTGAAGAATTGGAAAGAGGAGAGTGACAGAAAAATAA   229
A020R     ATGGAACTCTTTTCTTAGGCATTTTGAAGAATTGGAAAGAGGAGAGTGACAGAAAAATAA   229
N014R     ATGGAACTCTTTTCTTAGGCATTTTGAAGAATTGGAAAGAGGAGAGTGACAGAAAAATAA   228
N011R     ATGGAACTCTTTTCTTAGGCATTTTGAAGAATTGGAAAGAGGAGAGTGACAGAAAAATAA   228
N003R     ATGGAACTCTTTTCTTAGGCATTTTGAAGAATTGGAAAGAGGAGAGTGACAGAAAAATAA   229
A013R     ATGGAACTCTTTTCTTAGGCATTTTGAAGAATTGGAAAGAGGAGAGTGACAGAAAAATAA   229
A003R     ATGGAACTCTTTTCTTAGGCATTTTGAAGAATTGGAAAGAGGAGAGTGACAGAAAAATAA   225
A005R     ATGGAACTCTTTTCTTAGGCATTTTGAAGAATTGGAAAGAGGAGAGTGACAGAAAAATAA   227
A023R     ATGGAACTCTTTTCTTAGGCATTTTGAAGAATTGGAAAGAGGAGAGTGACAGAAAAATAA   227
N013R     ATGGAACTCTTTTCTTAGGCATTTTGAAGAATTGGAAAGAGGAGAGTGACAGAAAAATAA   229
N004R     ATGGAACTCTTTTCTTAGGCATTTTGAAGAATTGGAAAGAGGAGAGTGACAGAAAAATAA   230
A010R     ATGGAACTCTTTTCTTAGGCATTTTGAAGAATTGGAAAGAGGAGAGTGACAGAAAAATAA   226
N008R     ATGGAACTCTTTTCTTAGGCATTTTGAAGAATTGGAAAGAGGAGAGTGACAGAAAAATAA   230
A006R     ATGGAACTCTTTTCTTAGGCATTTTGAAGAATTGGAAAGAGGAGAGTGACAGAAAAATAA   229
A001R     ATGGAACTCTTTTCTTAGGCATTTTGAAGAATTGGAAAGAGGAGAGTGACAGAAAAATAA   211
A007R     ATGGAACTCTTTTCTTAGGCATTTTGAAGAATTGGAAAGAGGAGAGTGACAGAAAAATAA   229
          ************************************************************

N005      TGCAGAGCCAAATTGTCTCCTTTTACTTCAAACTTTTTAAAAACTTTAAAGATGACCAGA   257
N003      TGCAGAGCCAAATTGTCTCCTTTTACTTCAAACTTTTTAAAAACTTTAAAGATGACCAGA   258
A005      TGCAGAGCCAAATTGTCTCCTTTTACTTCAAACTTTTTAAAAACTTTAAAGATGACCAGA   256
A006      TGCAGAGCCAAATTGTCTCCTTTTACTTCAAACTTTTTAAAAACTTTAAAGATGACCAGA   258
A012      TGCAGAGCCAAATTGTCTCCTTTTACTTCAAACTTTTTAAAAACTTTAAAGATGACCAGA   257
A001      TGCAGAGCCAAATTGTCTCCTTTTACTTCAAACTTTTTAAAAACTTTAAAGATGACCAGA   254
N013      TGCAGAGCCAAATTGTCTCCTTTTACTTCAAACTTTTTAAAAACTTTAAAGATGACCAGA   257
N011      TGCAGAGCCAAATTGTCTCCTTTTACTTCAAACTTTTTAAAAACTTTAAAGATGACCAGA   254
N004      TGCAGAGCCAAATTGTCTCCTTTTACTTCAAACTTTTTAAAAACTTTAAAGATGACCAGA   255
N008      TGCAGAGCCAAATTGTCTCCTTTTACTTCAAACTTTTTAAAAACTTTAAAGATGACCAGA   255
N014      TGCAGAGCCAAATTGTCTCCTTTTACTTCAAACTTTTTAAAAACTTTAAAGATGACCAGA   259
A013      TGCAGAGCCAAATTGTCTCCTTTTACTTCAAACTTTTTAAAAACTTTAAAGATGACCAGA   256
A023      TGCAGAGCCAAATTGTCTCCTTTTACTTCAAACTTTTTAAAAACTTTAAAGATGACCAGA   257
A007      TGCAGAGCCAAATTGTCTCCTTTTACTTCAAACTTTTTAAAAACTTTAAAGATGACCAGA   256
A020      TGCAGAGCCAAATTGTCTCCTTTTACTTCAAACTTTTTAAAAACTTTAAAGATGACCAGA   255
A003      TGCAGAGCCAAATTGTCTCCTTTTACTTCAAACTTTTTAAAAACTTTAAAGATGACCAGA   257
A010      TGCAGAGCCAAATTGTCTCCTTTTACTTCAAACTTTTTAAAAACTTTAAAGATGACCAGA   255
N012      TGCAGAGCCAAATTGTCTCCTTTTACTTCAAACTTTTTAAAAACTTTAAAGATGACCAGA   257
N005R     TGCAGAGCCAAATTGTCTCCTTTTACTTCAAACTTTTTAAAAACTTTAAAGATGACCAGA   294
A012R     TGCAGAGCCAAATTGTCTCCTTTTACTTCAAACTTTTTAAAAACTTTAAAGATGACCAGA   286
N012R     TGCAGAGCCAAATTGTCTCCTTTTACTTCAAACTTTTTAAAAACTTTAAAGATGACCAGA   289
A020R     TGCAGAGCCAAATTGTCTCCTTTTACTTCAAACTTTTTAAAAACTTTAAAGATGACCAGA   289
N014R     TGCAGAGCCAAATTGTCTCCTTTTACTTCAAACTTTTTAAAAACTTTAAAGATGACCAGA   288
N011R     TGCAGAGCCAAATTGTCTCCTTTTACTTCAAACTTTTTAAAAACTTTAAAGATGACCAGA   288
N003R     TGCAGAGCCAAATTGTCTCCTTTTACTTCAAACTTTTTAAAAACTTTAAAGATGACCAGA   289
A013R     TGCAGAGCCAAATTGTCTCCTTTTACTTCAAACTTTTTAAAAACTTTAAAGATGACCAGA   289
A003R     TGCAGAGCCAAATTGTCTCCTTTTACTTCAAACTTTTTAAAAACTTTAAAGATGACCAGA   285
A005R     TGCAGAGCCAAATTGTCTCCTTTTACTTCAAACTTTTTAAAAACTTTAAAGATGACCAGA   287
A023R     TGCAGAGCCAAATTGTCTCCTTTTACTTCAAACTTTTTAAAAACTTTAAAGATGACCAGA   287
N013R     TGCAGAGCCAAATTGTCTCCTTTTACTTCAAACTTTTTAAAAACTTTAAAGATGACCAGA   289
N004R     TGCAGAGCCAAATTGTCTCCTTTTACTTCAAACTTTTTAAAAACTTTAAAGATGACCAGA   290
```

```
A010R     TGCAGAGCCAAATTGTCTCCTTTTACTTCAAACTTTTTAAAAACTTTAAAGATGACCAGA     286
N008R     TGCAGAGCCAAATTGTCTCCTTTTACTTCAAACTTTTTAAAAACTTTAAAGATGACCAGA     290
A006R     TGCAGAGCCAAATTGTCTCCTTTTACTTCAAACTTTTTAAAAACTTTAAAGATGACCAGA     289
A001R     TGCAGAGCCAAATTGTCTCCTTTTACTTCAAACTTTTTAAAAACTTTAAAGATGACCAGA     271
A007R     TGCAGAGCCAAATTGTCTCCTTTTACTTCAAACTTTTTAAAAACTTTAAAGATGACCAGA     289
          ************************************************************

N005      GCATCCAAAAGAGTGTGGAGACCATCAAGGAAGACATGAATGTCAAGTTTTTCAATAGCA     317
N003      GCATCCAAAAGAGTGTGGAGACCATCAAGGAAGACATGAATGTCAAGTTTTTCAATAGCA     318
A005      GCATCCAAAAGAGTGTGGAGACCATCAAGGAAGACATGAATGTCAAGTTTTTCAATAGCA     316
A006      GCATCCAAAAGAGTGTGGAGACCATCAAGGAAGACATGAATGTCAAGTTTTTCAATAGCA     318
A012      GCATCCAAAAGAGTGTGGAGACCATCAAGGAAGACATGAATGTCAAGTTTTTCAATAGCA     317
A001      GCATCCAAAAGAGTGTGGAGACCATCAAGGAAGACATGAATGTCAAGTTTTTCAATAGCA     314
N013      GCATCCAAAAGAGTGTGGAGACCATCAAGGAAGACATGAATGTCAAGTTTTTCAATAGCA     317
N011      GCATCCAAAAGAGTGTGGAGACCATCAAGGAAGACATGAATGTCAAGTTTTTCAATAGCA     314
N004      GCATCCAAAAGAGTGTGGAGACCATCAAGGAAGACATGAATGTCAAGTTTTTCAATAGCA     315
N008      GCATCCAAAAGAGTGTGGAGACCATCAAGGAAGACATGAATGTCAAGTTTTTCAATAGCA     315
N014      GCATCCAAAAGAGTGTGGAGACCATCAAGGAAGACATGAATGTCAAGTTTTTCAATAGCA     319
A013      GCATCCAAAAGAGTGTGGAGACCATCAAGGAAGACATGAATGTCAAGTTTTTCAATAGCA     316
A023      GCATCCAAAAGAGTGTGGAGACCATCAAGGAAGACATGAATGTCAAGTTTTTCAATAGCA     317
A007      GCATCCAAAAGAGTGTGGAGACCATCAAGGAAGACATGAATGTCAAGTTTTTCAATAGCA     316
A020      GCATCCAAAAGAGTGTGGAGACCATCAAGGAAGACATGAATGTCAAGTTTTTCAATAGCA     315
A003      GCATCCAAAAGAGTGTGGAGACCATCAAGGAAGACATGAATGTCAAGTTTTTCAATAGCA     317
A010      GCATCCAAAAGAGTGTGGAGACCATCAAGGAAGACATGAATGTCAAGTTTTTCAATAGCA     315
N012      GCATCCAAAAGAGTGTGGAGACCATCAAGGAAGACATGAATGTCAAGTTTTTCAATAGCA     317
N005R     GCATCCAAAAGAGTGTGGAGACCATCAAGGAAGACATGAATGTCAAGTTTTTCAATAGCA     354
A012R     GCATCCAAAAGAGTGTGGAGACCATCAAGGAAGACATGAATGTCAAGTTTTTCAATAGCA     346
N012R     GCATCCAAAAGAGTGTGGAGACCATCAAGGAAGACATGAATGTCAAGTTTTTCAATAGCA     349
A020R     GCATCCAAAAGAGTGTGGAGACCATCAAGGAAGACATGAATGTCAAGTTTTTCAATAGCA     349
N014R     GCATCCAAAAGAGTGTGGAGACCATCAAGGAAGACATGAATGTCAAGTTTTTCAATAGCA     348
N011R     GCATCCAAAAGAGTGTGGAGACCATCAAGGAAGACATGAATGTCAAGTTTTTCAATAGCA     348
N003R     GCATCCAAAAGAGTGTGGAGACCATCAAGGAAGACATGAATGTCAAGTTTTTCAATAGCA     349
A013R     GCATCCAAAAGAGTGTGGAGACCATCAAGGAAGACATGAATGTCAAGTTTTTCAATAGCA     349
A003R     GCATCCAAAAGAGTGTGGAGACCATCAAGGAAGACATGAATGTCAAGTTTTTCAATAGCA     345
A005R     GCATCCAAAAGAGTGTGGAGACCATCAAGGAAGACATGAATGTCAAGTTTTTCAATAGCA     347
A023R     GCATCCAAAAGAGTGTGGAGACCATCAAGGAAGACATGAATGTCAAGTTTTTCAATAGCA     347
N013R     GCATCCAAAAGAGTGTGGAGACCATCAAGGAAGACATGAATGTCAAGTTTTTCAATAGCA     349
N004R     GCATCCAAAAGAGTGTGGAGACCATCAAGGAAGACATGAATGTCAAGTTTTTCAATAGCA     350
A010R     GCATCCAAAAGAGTGTGGAGACCATCAAGGAAGACATGAATGTCAAGTTTTTCAATAGCA     346
N008R     GCATCCAAAAGAGTGTGGAGACCATCAAGGAAGACATGAATGTCAAGTTTTTCAATAGCA     350
A006R     GCATCCAAAAGAGTGTGGAGACCATCAAGGAAGACATGAATGTCAAGTTTTTCAATAGCA     349
A001R     GCATCCAAAAGAGTGTGGAGACCATCAAGGAAGACATGAATGTCAAGTTTTTCAATAGCA     331
A007R     GCATCCAAAAGAGTGTGGAGACCATCAAGGAAGACATGAATGTCAAGTTTTTCAATAGCA     349
          ************************************************************

N005      ACAAAAAGAAACGAGATGACTTCGAAAAGCTGACTAATTATTCGGTAACTGACTTGAATG     377
N003      ACAAAAAGAAACGAGATGACTTCGAAAAGCTGACTAATTATTCGGTAACTGACTTGAATG     378
A005      ACAAAAAGAAACGAGATGACTTCGAAAAGCTGACTAATTATTCGGTAACTGACTTGAATG     376
A006      ACAAAAAGAAACGAGATGACTTCGAAAAGCTGACTAATTATTCGGTAACTGACTTGAATG     378
A012      ACAAAAAGAAACGAGATGACTTCGAAAAGCTGACTAATTATTCGGTAACTGACTTGAATG     377
A001      ACAAAAAGAAACGAGATGACTTCGAAAAGCTGACTAATTATTCGGTAACTGACTTGAATG     374
N013      ACAAAAAGAAACGAGATGACTTCGAAAAGCTGACTAATTATTCGGTAACTGACTTGAATG     377
N011      ACAAAAAGAAACGAGATGACTTCGAAAAGCTGACTAATTATTCGGTAACTGACTTGAATG     374
N004      ACAAAAAGAAACGAGATGACTTCGAAAAGCTGACTAATTATTCGGTAACTGACTTGAATG     375
N008      ACAAAAAGAAACGAGATGACTTCGAAAAGCTGACTAATTATTCGGTAACTGACTTGAATG     375
N014      ACAAAAAGAAACGAGATGACTTCGAAAAGCTGACTAATTATTCGGTAACTGACTTGAATG     379
A013      ACAAAAAGAAACGAGATGACTTCGAAAAGCTGACTAATTATTCGGTAACTGACTTGAATG     376
A023      ACAAAAAGAAACGAGATGACTTCGAAAAGCTGACTAATTATTCGGTAACTGACTTGAATG     377
A007      ACAAAAAGAAACGAGATGACTTCGAAAAGCTGACTAATTATTCGGTAACTGACTTGAATG     376
A020      ACAAAAAGAAACGAGATGACTTCGAAAAGCTGACTAATTATTCGGTAACTGACTTGAATG     375
A003      ACAAAAAGAAACGAGATGACTTCGAAAAGCTGACTAATTATTCGGTAACTGACTTGAATG     377
A010      ACAAAAAGAAACGAGATGACTTCGAAAAGCTGACTAATTATTCGGTAACTGACTTGAATG     375
N012      ACAAAAAGAAACGAGATGACTTCGAAAAGCTGACTAATTATTCGGTAACTGACTTGAATG     377
```

```
N005R       ACAAAAAGAAACGAGATGACTTCGAAAAGCTGACTAATTATTCGGTAACTGACTTGAATG      414
A012R       ACAAAAAGAAACGAGATGACTTCGAAAAGCTGACTAATTATTCGGTAACTGACTTGAATG      406
N012R       ACAAAAAGAAACGAGATGACTTCGAAAAGCTGACTAATTATTCGGTAACTGACTTGAATG      409
A020R       ACAAAAAGAAACGAGATGACTTCGAAAAGCTGACTAATTATTCGGTAACTGACTTGAATG      409
N014R       ACAAAAAGAAACGAGATGACTTCGAAAAGCTGACTAATTATTCGGTAACTGACTTGAATG      408
N011R       ACAAAAAGAAACGAGATGACTTCGAAAAGCTGACTAATTATTCGGTAACTGACTTGAATG      408
N003R       ACAAAAAGAAACGAGATGACTTCGAAAAGCTGACTAATTATTCGGTAACTGACTTGAATG      409
A013R       ACAAAAAGAAACGAGATGACTTCGAAAAGCTGACTAATTATTCGGTAACTGACTTGAATG      409
A003R       ACAAAAAGAAACGAGATGACTTCGAAAAGCTGACTAATTATTCGGTAACTGACTTGAATG      405
A005R       ACAAAAAGAAACGAGATGACTTCGAAAAGCTGACTAATTATTCGGTAACTGACTTGAATG      407
A023R       ACAAAAAGAAACGAGATGACTTCGAAAAGCTGACTAATTATTCGGTAACTGACTTGAATG      407
N013R       ACAAAAAGAAACGAGATGACTTCGAAAAGCTGACTAATTATTCGGTAACTGACTTGAATG      409
N004R       ACAAAAAGAAACGAGATGACTTCGAAAAGCTGACTAATTATTCGGTAACTGACTTGAATG      410
A010R       ACAAAAAGAAACGAGATGACTTCGAAAAGCTGACTAATTATTCGGTAACTGACTTGAATG      406
N008R       ACAAAAAGAAACGAGATGACTTCGAAAAGCTGACTAATTATTCGGTAACTGACTTGAATG      410
A006R       ACAAAAAGAAACGAGATGACTTCGAAAAGCTGACTAATTATTCGGTAACTGACTTGAATG      409
A001R       ACAAAAAGAAACGAGATGACTTCGAAAAGCTGACTAATTATTCGGTAACTGACTTGAATG      391
A007R       ACAAAAAGAAACGAGATGACTTCGAAAAGCTGACTAATTATTCGGTAACTGACTTGAATG      409
            ************************************************************

N005        TCCAACGCAAAGCAATACATGAACTCATCCAAGTGATGGCTGAACTGTCGCCAGCAGCTA      437
N003        TCCAACGCAAAGCAATACATGAACTCATCCAAGTGATGGCTGAACTGTCGCCAGCAGCTA      438
A005        TCCAACGCAAAGCAATACATGAACTCATCCAAGTGATGGCTGAACTGTCGCCAGCAGCTA      436
A006        TCCAACGCAAAGCAATACATGAACTCATCCAAGTGATGGCTGAACTGTCGCCAGCAGCTA      438
A012        TCCAACGCAAAGCAATACATGAACTCATCCAAGTGATGGCTGAACTGTCGCCAGCAGCTA      437
A001        TCCAACGCAAAGCAATACATGAACTCATCCAAGTGATGGCTGAACTGTCGCCAGCAGCTA      434
N013        TCCAACGCAAAGCAATACATGAACTCATCCAAGTGATGGCTGAACTGTCGCCAGCAGCTA      437
N011        TCCAACGCAAAGCAATACATGAACTCATCCAAGTGATGGCTGAACTGTCGCCAGCAGCTA      434
N004        TCCAACGCAAAGCAATACATGAACTCATCCAAGTGATGGCTGAACTGTCGCCAGCAGCTA      435
N008        TCCAACGCAAAGCAATACATGAACTCATCCAAGTGATGGCTGAACTGTCGCCAGCAGCTA      435
N014        TCCAACGCAAAGCAATACATGAACTCATCCAAGTGATGGCTGAACTGTCGCCAGCAGCTA      439
A013        TCCAACGCAAAGCAATACATGAACTCATCCAAGTGATGGCTGAACTGTCGCCAGCAGCTA      436
A023        TCCAACGCAAAGCAATACATGAACTCATCCAAGTGATGGCTGAACTGTCGCCAGCAGCTA      437
A007        TCCAACGCAAAGCAATACATGAACTCATCCAAGTGATGGCTGAACTGTCGCCAGCAGCTA      436
A020        TCCAACGCAAAGCAATACATGAACTCATCCAAGTGATGGCTGAACTGTCGCCAGCAGCTA      435
A003        TCCAACGCAAAGCAATACATGAACTCATCCAAGTGATGGCTGAACTGTCGCCAGCAGCTA      437
A010        TCCAACGCAAAGCAATACATGAACTCATCCAAGTGATGGCTGAACTGTCGCCAGCAGCTA      435
N012        TCCAACGCAAAGCAATACATGAACTCATCCAAGTGATGGCTGAACTGTCGCCAGCAGCTA      437
N005R       TCCAACGCAAAGCAATACATGAACTCATCCAAGTGATGGCTGAACTGTCGCCAGCAGCTA      474
A012R       TCCAACGCAAAGCAATACATGAACTCATCCAAGTGATGGCTGAACTGTCGCCAGCAGCTA      466
N012R       TCCAACGCAAAGCAATACATGAACTCATCCAAGTGATGGCTGAACTGTCGCCAGCAGCTA      469
A020R       TCCAACGCAAAGCAATACATGAACTCATCCAAGTGATGGCTGAACTGTCGCCAGCAGCTA      469
N014R       TCCAACGCAAAGCAATACATGAACTCATCCAAGTGATGGCTGAACTGTCGCCAGCAGCTA      468
N011R       TCCAACGCAAAGCAATACATGAACTCATCCAAGTGATGGCTGAACTGTCGCCAGCAGCTA      468
N003R       TCCAACGCAAAGCAATACATGAACTCATCCAAGTGATGGCTGAACTGTCGCCAGCAGCTA      469
A013R       TCCAACGCAAAGCAATACATGAACTCATCCAAGTGATGGCTGAACTGTCGCCAGCAGCTA      469
A003R       TCCAACGCAAAGCAATACATGAACTCATCCAAGTGATGGCTGAACTGTCGCCAGCAGCTA      465
A005R       TCCAACGCAAAGCAATACATGAACTCATCCAAGTGATGGCTGAACTGTCGCCAGCAGCTA      467
A023R       TCCAACGCAAAGCAATACATGAACTCATCCAAGTGATGGCTGAACTGTCGCCAGCAGCTA      467
N013R       TCCAACGCAAAGCAATACATGAACTCATCCAAGTGATGGCTGAACTGTCGCCAGCAGCTA      469
N004R       TCCAACGCAAAGCAATACATGAACTCATCCAAGTGATGGCTGAACTGTCGCCAGCAGCTA      470
A010R       TCCAACGCAAAGCAATACATGAACTCATCCAAGTGATGGCTGAACTGTCGCCAGCAGCTA      466
N008R       TCCAACGCAAAGCAATACATGAACTCATCCAAGTGATGGCTGAACTGTCGCCAGCAGCTA      470
A006R       TCCAACGCAAAGCAATACATGAACTCATCCAAGTGATGGCTGAACTGTCGCCAGCAGCTA      469
A001R       TCCAACGCAAAGCAATACATGAACTCATCCAAGTGATGGCTGAACTGTCGCCAGCAGCTA      451
A007R       TCCAACGCAAAGCAATACATGAACTCATCCAAGTGATGGCTGAACTGTCGCCAGCAGCTA      469
            ************************************************************

N005        AAAC-AGGGAAGCGAAAAAGGAGTCAGATGCTGTTTCGAGGTCGAAGAGCATCCCAGTAA      496
N003        AAAC-AGGGAAGCGAAAAAGGAGTCAGATGCTGTTTCGAGGTCGAAGAGCATCCCAGAAT      497
A005        AAAC-AGGGAAGCGAAAAAGGAGTCAGATGCTGTTTCGAGGTCGAAGAGCATCCCAGAAG      495
A006        AAAC-AGGGAAGCGAAAAAGGAGTCAGATGCTGTTTCGAGGTCGAAGAGCATCCCAGTAT      497
A012        AAAC-AGGGAAGCGAAAAAGGAGTCAGATGCTGTTTCGAGGTCGAAGAGCATCCCAGATT      496
```

```
A001     AAAC-AGGGAAGCGAAAAAGGAGTCAGATGCTGTTTCGAGGTCAAAGAGCATCCCAGATT   493
N013     AAAC-AGGGAAGCGAAAAAGGAGTCAGATGCTGTTTCGAGGTCGAAGAGCATCCCAGTAT   496
N011     AAAC-AGGGAAGCGAAAAAGGAGTCAGATGCTGTTTCGAGGTCGAAGAGCATCCCAGTAT   493
N004     AAAC-AGGGAAGCGAAAAAGGAGTCAGATGCTGTTTCGAGGTCGAAGAGCATCCCAGTAT   494
N008     AAAC-AGGGAAGCGAAAAAGGAGTCAGATGCTGTTTCGAGGTCGAAGAGCATCCCAGATT   494
N014     AAAC-AGGGAAGCGAAAAAGGAGTCAGATGCTGTTTCGAGGTCGAAGAGCATCCCAGTAT   498
A013     AAAC-AGGGAAGCGAAAAAGGAGTCAGATGCTGTTTCGAGGTCGAAGAGCATCCCAGTAA   495
A023     AAAC-AGGGAAGCGAAAAAGGAGTCAGATGCTGTTTCGAGGTCGAAGAGCATCCCAGTAA   496
A007     AAAC-AGGGAAGCGAAAAAGGAGTCAGATGCTGTTTCGAGGTCGAAGAGCATCCCAGTAT   495
A020     AAAC-AGGGAAGCGAAAAAGGAGTCAGATGCTGTTTCGAGGTCAAAGAGCATCCCAGTAA   494
A003     AAAC-AGGGAAGCGAAAAAGGAGTCAGATGCTGTTTCGAGGTCGAAGAGCATCCCAGTAT   496
A010     AAAC-AGGGAAGCGAAAAAGGAGTCAGATGCTGTTTCGAGGTCGAAGAGCATCCCAGTAT   494
N012     AAAC-AGGGAAGCGAAAAAGGAGTCAGATGCTGTTTCGAGGTCGAAGAGCATCCCAGTAT   496
N005R    AACCGAGGGAAACGAATCAAGAGCCAACCCCAGTT------------------------A   510
A012R    AAAC-AGGGAAGCGAAAAAGGAGTCCAGATGCGTC------------------------A   501
N012R    AAAC-AGGGAAGCGAAAAAGGAGTCCAGATGCCGT------------------------C   504
A020R    AAAC-AGGGAAGCGAAAAAGGAGTC-AGATGCCGT------------------------C   503
N014R    AAAC-AGGGAAGCGAAAAAGGAGTC-AGATGCCGA------------------------C   502
N011R    AAAC-AGGGAAGCGAAAAAGGAGTCCAGATGCGTC------------------------G   503
N003R    AAAC-AGGGAAGCGAAAAAGGAGTC-AGATGCGTC------------------------A   503
A013R    AAAC-AGGGAAGCGAAAAAGGAGTCCAGATGCGTC------------------------G   504
A003R    AAAC-AGGGAAGCGAAAAAGGAGTC-AGATGCGTC------------------------G   499
A005R    AAAC-AGGGAAGCGAAAAAGGAGTC-AGATGCGAC------------------------G   501
A023R    AAAC-AGGGAAGCGAAAAAGGAGTC-AGATGCTAC------------------------A   501
N013R    AAAC-AGGGAAGCGAAAAAGGAGTC-AGATGCGAC------------------------G   503
N004R    AAAC-AGGGAAGCGAAAAAGGAGTC-AGATGCGAC------------------------G   504
A010R    AAAC-AGGGAAGCGAAAAAGGAGTC-AGATGCGTC------------------------A   500
N008R    AAAC-AGGGAAGCGAAAAAGGAGTC-AGATGCGTC------------------------T   504
A006R    AAAC-AGGGAAGCGAAAAAGGAGTC-AGATGCGTA------------------------G   503
A001R    AAAC-AGGGAAGCGAAAAAGGAGTC-AGATGCGTC------------------------G   485
A007R    AAAC-AGGGAAGCGAAAAAGGAGTC-AGATGCTTC------------------------G   503
```

SET 2

```
N001F    ---------------------------------------------------TCGTTTTGG   9
A009F    ---------------------------------------------------TCGTTTTGG   9
A002F    -------------------------------------------GCTCTGCATCGTTTTGG   17
A004F    -------------------------------------------GCTCTGCATCGTTTTGG   17
A008F    -------------------------------------------GCTCTGCATCGTTTTGG   17
A011F    -------------------------------------------GCTCTGCATCGTTTTGG   17
A014F    -------------------------------------------GCTCTGCATCGTTTTGG   17
A015F    -------------------------------------------GCTCTGCATCGTTTTGG   17
A016F    -------------------------------------------GCTCTGCATCGTTTTGG   17
A017F    -------------------------------------------GCTCTGCATCGTTTTGG   17
A018F    -------------------------------------------GCTCTGCATCGTTTTGG   17
N006F    -------------------------------------------GCTCTGCATCGTTTTGG   17
N007F    -------------------------------------------GCTCTGCATCGTTTTGG   17
N009F    -------------------------------------------GCTCTGCATCGTTTTGG   17
N010F    -------------------------------------------GCTCTGCATCGTTTTGG   17
A002R    ----GGAAACGATGAAATATACAAGTTATATCTTGGCTTTTCAGCTCTGCATCGTTTTGG   56
A008R    ---CGGAAACGATGAAATATACAAGTTATATCTTGGCTTTTCAGCTCTGCATCGTTTTGG   57
A015R    TCTCGGAAACGATGAAATATACAAGTTATATCTTGGCTTTTCAGCTCTGCATCGTTTTGG   60
A016R    TCTCGGAAACGATGAAATATACAAGTTATATCTTGGCTTTTCAGCTCTGCATCGTTTTGG   60
A017R    ---CGGAAACGATGAAATATACAAGTTATATCTTGGCTTTTCAGCTCTGCATCGTTTTGG   57
A018R    ----GGAAACGATGAAATATACAAGTTATATCTTGGCTTTTCAGCTCTGCATCGTTTTGG   56
N006R    ---CGGAAACGATGAAATATACAAGTTATATCTTGGCTTTTCAGCTCTGCATCGTTTTGG   57
N007R    ---CGGAAACGATGAAATATACAAGTTATATCTTGGCTTTTCAGCTCTGCATCGTTTTGG   57
N009R    ----GGAAACGATGAAATATACAAGTTATATCTTGGCTTTTCAGCTCTGCATCGTTTTGG   56
N010R    -CTCGGAAACGATGAAATATACAAGTTATATCTTGGCTTTTCAGCTCTGCATCGTTTTGG   59
A004R    -CTCGGAAACGATGAAATATACAAGTTATATCTTGGCTTTTCAGCTCTGCATCGTTTTGG   59
A009R    TCTCGGAAACGATGAAATATACAAGTTATATCTTGGCTTTTCAGCTCTGCATCGTTTTGG   60
A011R    TCTCGGAAACGATGAAATATACAAGTTATATCTTGGCTTTTCAGCTCTGCATCGTTTTGG   60
```

```
A014R      --TCGGAAACGATGAAATATACAAGTTATATCTTGGCTTTTCAGCTCTGCATCGTTTTGG      58
N001R      --TCGGAAACGATGAAATATACAAGTTATATCTTGGCTTTTCAGCTCTGCATCGTTTTGG      58
                                                                *********

N001F      GTTCTCTTGGCTGTTACTGCCAGGACCCATATGTAAAAGAAGCAGAAAACCTTAAGAAAT      69
A009F      GTTCTCTTGGCTGTTACTGCCAGGACCCATATGTAAAAGAAGCAGAAAACCTTAAGAAAT      69
A002F      GTTCTCTTGGCTGTTACTGCCAGGACCCATATGTAAAAGAAGCAGAAAACCTTAAGAAAT      77
A004F      GTTCTCTTGGCTGTTACTGCCAGGACCCATATGTAAAAGAAGCAGAAAACCTTAAGAAAT      77
A008F      GTTCTCTTGGCTGTTACTGCCAGGACCCATATGTAAAAGAAGCAGAAAACCTTAAGAAAT      77
A011F      GTTCTCTTGGCTGTTACTGCCAGGACCCATATGTAAAAGAAGCAGAAAACCTTAAGAAAT      77
A014F      GTTCTCTTGGCTGTTACTGCCAGGACCCATATGTAAAAGAAGCAGAAAACCTTAAGAAAT      77
A015F      GTTCTCTTGGCTGTTACTGCCAGGACCCATATGTAAAAGAAGCAGAAAACCTTAAGAAAT      77
A016F      GTTCTCTTGGCTGTTACTGCCAGGACCCATATGTAAAAGAAGCAGAAAACCTTAAGAAAT      77
A017F      GTTCTCTTGGCTGTTACTGCCAGGACCCATATGTAAAAGAAGCAGAAAACCTTAAGAAAT      77
A018F      GTTCTCTTGGCTGTTACTGCCAGGACCCATATGTAAAAGAAGCAGAAAACCTTAAGAAAT      77
N006F      GTTCTCTTGGCTGTTACTGCCAGGACCCATATGTAAAAGAAGCAGAAAACCTTAAGAAAT      77
N007F      GTTCTCTTGGCTGTTACTGCCAGGACCCATATGTAAAAGAAGCAGAAAACCTTAAGAAAT      77
N009F      GTTCTCTTGGCTGTTACTGCCAGGACCCATATGTAAAAGAAGCAGAAAACCTTAAGAAAT      77
N010F      GTTCTCTTGGCTGTTACTGCCAGGACCCATATGTAAAAGAAGCAGAAAACCTTAAGAAAT      77
A002R      GTTCTCTTGGCTGTTACTGCCAGGACCCATATGTAAAAGAAGCAGAAAACCTTAAGAAAT      116
A008R      GTTCTCTTGGCTGTTACTGCCAGGACCCATATGTAAAAGAAGCAGAAAACCTTAAGAAAT      117
A015R      GTTCTCTTGGCTGTTACTGCCAGGACCCATATGTAAAAGAAGCAGAAAACCTTAAGAAAT      120
A016R      GTTCTCTTGGCTGTTACTGCCAGGACCCATATGTAAAAGAAGCAGAAAACCTTAAGAAAT      120
A017R      GTTCTCTTGGCTGTTACTGCCAGGACCCATATGTAAAAGAAGCAGAAAACCTTAAGAAAT      117
A018R      GTTCTCTTGGCTGTTACTGCCAGGACCCATATGTAAAAGAAGCAGAAAACCTTAAGAAAT      116
N006R      GTTCTCTTGGCTGTTACTGCCAGGACCCATATGTAAAAGAAGCAGAAAACCTTAAGAAAT      117
N007R      GTTCTCTTGGCTGTTACTGCCAGGACCCATATGTAAAAGAAGCAGAAAACCTTAAGAAAT      117
N009R      GTTCTCTTGGCTGTTACTGCCAGGACCCATATGTAAAAGAAGCAGAAAACCTTAAGAAAT      116
N010R      GTTCTCTTGGCTGTTACTGCCAGGACCCATATGTAAAAGAAGCAGAAAACCTTAAGAAAT      119
A004R      GTTCTCTTGGCTGTTACTGCCAGGACCCATATGTAAAAGAAGCAGAAAACCTTAAGAAAT      119
A009R      GTTCTCTTGGCTGTTACTGCCAGGACCCATATGTAAAAGAAGCAGAAAACCTTAAGAAAT      120
A011R      GTTCTCTTGGCTGTTACTGCCAGGACCCATATGTAAAAGAAGCAGAAAACCTTAAGAAAT      120
A014R      GTTCTCTTGGCTGTTACTGCCAGGACCCATATGTAAAAGAAGCAGAAAACCTTAAGAAAT      118
N001R      GTTCTCTTGGCTGTTACTGCCAGGACCCATATGTAAAAGAAGCAGAAAACCTTAAGAAAT      118
           ************************************************************

N001F      ATTTTAATGCAGGTCATTCAGATGTAGCGGATAATGGAACTCTTTTCTTAGGCATTTTGA      129
A009F      ATTTTAATGCAGGTCATTCAGATGTAGCGGATAATGGAACTCTTTTCTTAGGCATTTTGA      129
A002F      ATTTTAATGCAGGTCATTCAGATGTAGCGGATAATGGAACTCTTTTCTTAGGCATTTTGA      137
A004F      ATTTTAATGCAGGTCATTCAGATGTAGCGGATAATGGAACTCTTTTCTTAGGCATTTTGA      137
A008F      ATTTTAATGCAGGTCATTCAGATGTAGCGGATAATGGAACTCTTTTCTTAGGCATTTTGA      137
A011F      ATTTTAATGCAGGTCATTCAGATGTAGCGGATAATGGAACTCTTTTCTTAGGCATTTTGA      137
A014F      ATTTTAATGCAGGTCATTCAGATGTAGCGGATAATGGAACTCTTTTCTTAGGCATTTTGA      137
A015F      ATTTTAATGCAGGTCATTCAGATGTAGCGGATAATGGAACTCTTTTCTTAGGCATTTTGA      137
A016F      ATTTTAATGCAGGTCATTCAGATGTAGCGGATAATGGAACTCTTTTCTTAGGCATTTTGA      137
A017F      ATTTTAATGCAGGTCATTCAGATGTAGCGGATAATGGAACTCTTTTCTTAGGCATTTTGA      137
A018F      ATTTTAATGCAGGTCATTCAGATGTAGCGGATAATGGAACTCTTTTCTTAGGCATTTTGA      137
N006F      ATTTTAATGCAGGTCATTCAGATGTAGCGGATAATGGAACTCTTTTCTTAGGCATTTTGA      137
N007F      ATTTTAATGCAGGTCATTCAGATGTAGCGGATAATGGAACTCTTTTCTTAGGCATTTTGA      137
N009F      ATTTTAATGCAGGTCATTCAGATGTAGCGGATAATGGAACTCTTTTCTTAGGCATTTTGA      137
N010F      ATTTTAATGCAGGTCATTCAGATGTAGCGGATAATGGAACTCTTTTCTTAGGCATTTTGA      137
A002R      ATTTTAATGCAGGTCATTCAGATGTAGCGGATAATGGAACTCTTTTCTTAGGCATTTTGA      176
A008R      ATTTTAATGCAGGTCATTCAGATGTAGCGGATAATGGAACTCTTTTCTTAGGCATTTTGA      177
A015R      ATTTTAATGCAGGTCATTCAGATGTAGCGGATAATGGAACTCTTTTCTTAGGCATTTTGA      180
A016R      ATTTTAATGCAGGTCATTCAGATGTAGCGGATAATGGAACTCTTTTCTTAGGCATTTTGA      180
A017R      ATTTTAATGCAGGTCATTCAGATGTAGCGGATAATGGAACTCTTTTCTTAGGCATTTTGA      177
A018R      ATTTTAATGCAGGTCATTCAGATGTAGCGGATAATGGAACTCTTTTCTTAGGCATTTTGA      176
N006R      ATTTTAATGCAGGTCATTCAGATGTAGCGGATAATGGAACTCTTTTCTTAGGCATTTTGA      177
N007R      ATTTTAATGCAGGTCATTCAGATGTAGCGGATAATGGAACTCTTTTCTTAGGCATTTTGA      177
N009R      ATTTTAATGCAGGTCATTCAGATGTAGCGGATAATGGAACTCTTTTCTTAGGCATTTTGA      176
N010R      ATTTTAATGCAGGTCATTCAGATGTAGCGGATAATGGAACTCTTTTCTTAGGCATTTTGA      179
A004R      ATTTTAATGCAGGTCATTCAGATGTAGCGGATAATGGAACTCTTTTCTTAGGCATTTTGA      179
A009R      ATTTTAATGCAGGTCATTCAGATGTAGCGGATAATGGAACTCTTTTCTTAGGCATTTTGA      180
```

```
A011R      ATTTTAATGCAGGTCATTCAGATGTAGCGGATAATGGAACTCTTTTCTTAGGCATTTTGA      180
A014R      ATTTTAATGCAGGTCATTCAGATGTAGCGGATAATGGAACTCTTTTCTTAGGCATTTTGA      178
N001R      ATTTTAATGCAGGTCATTCAGATGTAGCGGATAATGGAACTCTTTTCTTAGGCATTTTGA      178
           ************************************************************

N001F      AGAATTGGAAAGAGGAGAGTGACAGAAAAATAATGCAGAGCCAAATTGTCTCCTTTTACT      189
A009F      AGAATTGGAAAGAGGAGAGTGACAGAAAAATAATGCAGAGCCAAATTGTCTCCTTTTACT      189
A002F      AGAATTGGAAAGAGGAGAGTGACAGAAAAATAATGCAGAGCCAAATTGTCTCCTTTTACT      197
A004F      AGAATTGGAAAGAGGAGAGTGACAGAAAAATAATGCAGAGCCAAATTGTCTCCTTTTACT      197
A008F      AGAATTGGAAAGAGGAGAGTGACAGAAAAATAATGCAGAGCCAAATTGTCTCCTTTTACT      197
A011F      AGAATTGGAAAGAGGAGAGTGACAGAAAAATAATGCAGAGCCAAATTGTCTCCTTTTACT      197
A014F      AGAATTGGAAAGAGGAGAGTGACAGAAAAATAATGCAGAGCCAAATTGTCTCCTTTTACT      197
A015F      AGAATTGGAAAGAGGAGAGTGACAGAAAAATAATGCAGAGCCAAATTGTCTCCTTTTACT      197
A016F      AGAATTGGAAAGAGGAGAGTGACAGAAAAATAATGCAGAGCCAAATTGTCTCCTTTTACT      197
A017F      AGAATTGGAAAGAGGAGAGTGACAGAAAAATAATGCAGAGCCAAATTGTCTCCTTTTACT      197
A018F      AGAATTGGAAAGAGGAGAGTGACAGAAAAATAATGCAGAGCCAAATTGTCTCCTTTTACT      197
N006F      AGAATTGGAAAGAGGAGAGTGACAGAAAAATAATGCAGAGCCAAATTGTCTCCTTTTACT      197
N007F      AGAATTGGAAAGAGGAGAGTGACAGAAAAATAATGCAGAGCCAAATTGTCTCCTTTTACT      197
N009F      AGAATTGGAAAGAGGAGAGTGACAGAAAAATAATGCAGAGCCAAATTGTCTCCTTTTACT      197
N010F      AGAATTGGAAAGAGGAGAGTGACAGAAAAATAATGCAGAGCCAAATTGTCTCCTTTTACT      197
A002R      AGAATTGGAAAGAGGAGAGTGACAGAAAAATAATGCAGAGCCAAATTGTCTCCTTTTACT      236
A008R      AGAATTGGAAAGAGGAGAGTGACAGAAAAATAATGCAGAGCCAAATTGTCTCCTTTTACT      237
A015R      AGAATTGGAAAGAGGAGAGTGACAGAAAAATAATGCAGAGCCAAATTGTCTCCTTTTACT      240
A016R      AGAATTGGAAAGAGGAGAGTGACAGAAAAATAATGCAGAGCCAAATTGTCTCCTTTTACT      240
A017R      AGAATTGGAAAGAGGAGAGTGACAGAAAAATAATGCAGAGCCAAATTGTCTCCTTTTACT      237
A018R      AGAATTGGAAAGAGGAGAGTGACAGAAAAATAATGCAGAGCCAAATTGTCTCCTTTTACT      236
N006R      AGAATTGGAAAGAGGAGAGTGACAGAAAAATAATGCAGAGCCAAATTGTCTCCTTTTACT      237
N007R      AGAATTGGAAAGAGGAGAGTGACAGAAAAATAATGCAGAGCCAAATTGTCTCCTTTTACT      237
N009R      AGAATTGGAAAGAGGAGAGTGACAGAAAAATAATGCAGAGCCAAATTGTCTCCTTTTACT      236
N010R      AGAATTGGAAAGAGGAGAGTGACAGAAAAATAATGCAGAGCCAAATTGTCTCCTTTTACT      239
A004R      AGAATTGGAAAGAGGAGAGTGACAGAAAAATAATGCAGAGCCAAATTGTCTCCTTTTACT      239
A009R      AGAATTGGAAAGAGGAGAGTGACAGAAAAATAATGCAGAGCCAAATTGTCTCCTTTTACT      240
A011R      AGAATTGGAAAGAGGAGAGTGACAGAAAAATAATGCAGAGCCAAATTGTCTCCTTTTACT      240
A014R      AGAATTGGAAAGAGGAGAGTGACAGAAAAATAATGCAGAGCCAAATTGTCTCCTTTTACT      238
N001R      AGAATTGGAAAGAGGAGAGTGACAGAAAAATAATGCAGAGCCAAATTGTCTCCTTTTACT      238
           ************************************************************

N001F      TCAAACTTTTTAAAAACTTTAAAGATGACCAGAGCATCCAAAAGAGTGTGGAGACCATCA      249
A009F      TCAAACTTTTTAAAAACTTTAAAGATGACCAGAGCATCCAAAAGAGTGTGGAGACCATCA      249
A002F      TCAAACTTTTTAAAAACTTTAAAGATGACCAGAGCATCCAAAAGAGTGTGGAGACCATCA      257
A004F      TCAAACTTTTTAAAAACTTTAAAGATGACCAGAGCATCCAAAAGAGTGTGGAGACCATCA      257
A008F      TCAAACTTTTTAAAAACTTTAAAGATGACCAGAGCATCCAAAAGAGTGTGGAGACCATCA      257
A011F      TCAAACTTTTTAAAAACTTTAAAGATGACCAGAGCATCCAAAAGAGTGTGGAGACCATCA      257
A014F      TCAAACTTTTTAAAAACTTTAAAGATGACCAGAGCATCCAAAAGAGTGTGGAGACCATCA      257
A015F      TCAAACTTTTTAAAAACTTTAAAGATGACCAGAGCATCCAAAAGAGTGTGGAGACCATCA      257
A016F      TCAAACTTTTTAAAAACTTTAAAGATGACCAGAGCATCCAAAAGAGTGTGGAGACCATCA      257
A017F      TCAAACTTTTTAAAAACTTTAAAGATGACCAGAGCATCCAAAAGAGTGTGGAGACCATCA      257
A018F      TCAAACTTTTTAAAAACTTTAAAGATGACCAGAGCATCCAAAAGAGTGTGGAGACCATCA      257
N006F      TCAAACTTTTTAAAAACTTTAAAGATGACCAGAGCATCCAAAAGAGTGTGGAGACCATCA      257
N007F      TCAAACTTTTTAAAAACTTTAAAGATGACCAGAGCATCCAAAAGAGTGTGGAGACCATCA      257
N009F      TCAAACTTTTTAAAAACTTTAAAGATGACCAGAGCATCCAAAAGAGTGTGGAGACCATCA      257
N010F      TCAAACTTTTTAAAAACTTTAAAGATGACCAGAGCATCCAAAAGAGTGTGGAGACCATCA      257
A002R      TCAAACTTTTTAAAAACTTTAAAGATGACCAGAGCATCCAAAAGAGTGTGGAGACCATCA      296
A008R      TCAAACTTTTTAAAAACTTTAAAGATGACCAGAGCATCCAAAAGAGTGTGGAGACCATCA      297
A015R      TCAAACTTTTTAAAAACTTTAAAGATGACCAGAGCATCCAAAAGAGTGTGGAGACCATCA      300
A016R      TCAAACTTTTTAAAAACTTTAAAGATGACCAGAGCATCCAAAAGAGTGTGGAGACCATCA      300
A017R      TCAAACTTTTTAAAAACTTTAAAGATGACCAGAGCATCCAAAAGAGTGTGGAGACCATCA      297
A018R      TCAAACTTTTTAAAAACTTTAAAGATGACCAGAGCATCCAAAAGAGTGTGGAGACCATCA      296
N006R      TCAAACTTTTTAAAAACTTTAAAGATGACCAGAGCATCCAAAAGAGTGTGGAGACCATCA      297
N007R      TCAAACTTTTTAAAAACTTTAAAGATGACCAGAGCATCCAAAAGAGTGTGGAGACCATCA      297
N009R      TCAAACTTTTTAAAAACTTTAAAGATGACCAGAGCATCCAAAAGAGTGTGGAGACCATCA      296
N010R      TCAAACTTTTTAAAAACTTTAAAGATGACCAGAGCATCCAAAAGAGTGTGGAGACCATCA      299
A004R      TCAAACTTTTTAAAAACTTTAAAGATGACCAGAGCATCCAAAAGAGTGTGGAGACCATCA      299
```

```
A009R      TCAAACTTTTTAAAAACTTTAAAGATGACCAGAGCATCCAAAAGAGTGTGGAGACCATCA      300
A011R      TCAAACTTTTTAAAAACTTTAAAGATGACCAGAGCATCCAAAAGAGTGTGGAGACCATCA      300
A014R      TCAAACTTTTTAAAAACTTTAAAGATGACCAGAGCATCCAAAAGAGTGTGGAGACCATCA      298
N001R      TCAAACTTTTTAAAAACTTTAAAGATGACCAGAGCATCCAAAAGAGTGTGGAGACCATCA      298
           ************************************************************

N001F      AGGAAGACATGAATGTCAAGTTTTTCAATAGCAACAAAAAGAAACGAGATGACTTCGAAA      309
A009F      AGGAAGACATGAATGTCAAGTTTTTCAATAGCAACAAAAAGAAACGAGATGACTTCGAAA      309
A002F      AGGAAGACATGAATGTCAAGTTTTTCAATAGCAACAAAAAGAAACGAGATGACTTCGAAA      317
A004F      AGGAAGACATGAATGTCAAGTTTTTCAATAGCAACAAAAAGAAACGAGATGACTTCGAAA      317
A008F      AGGAAGACATGAATGTCAAGTTTTTCAATAGCAACAAAAAGAAACGAGATGACTTCGAAA      317
A011F      AGGAAGACATGAATGTCAAGTTTTTCAATAGCAACAAAAAGAAACGAGATGACTTCGAAA      317
A014F      AGGAAGACATGAATGTCAAGTTTTTCAATAGCAACAAAAAGAAACGAGATGACTTCGAAA      317
A015F      AGGAAGACATGAATGTCAAGTTTTTCAATAGCAACAAAAAGAAACGAGATGACTTCGAAA      317
A016F      AGGAAGACATGAATGTCAAGTTTTTCAATAGCAACAAAAAGAAACGAGATGACTTCGAAA      317
A017F      AGGAAGACATGAATGTCAAGTTTTTCAATAGCAACAAAAAGAAACGAGATGACTTCGAAA      317
A018F      AGGAAGACATGAATGTCAAGTTTTTCAATAGCAACAAAAAGAAACGAGATGACTTCGAAA      317
N006F      AGGAAGACATGAATGTCAAGTTTTTCAATAGCAACAAAAAGAAACGAGATGACTTCGAAA      317
N007F      AGGAAGACATGAATGTCAAGTTTTTCAATAGCAACAAAAAGAAACGAGATGACTTCGAAA      317
N009F      AGGAAGACATGAATGTCAAGTTTTTCAATAGCAACAAAAAGAAACGAGATGACTTCGAAA      317
N010F      AGGAAGACATGAATGTCAAGTTTTTCAATAGCAACAAAAAGAAACGAGATGACTTCGAAA      317
A002R      AGGAAGACATGAATGTCAAGTTTTTCAATAGCAACAAAAAGAAACGAGATGACTTCGAAA      356
A008R      AGGAAGACATGAATGTCAAGTTTTTCAATAGCAACAAAAAGAAACGAGATGACTTCGAAA      357
A015R      AGGAAGACATGAATGTCAAGTTTTTCAATAGCAACAAAAAGAAACGAGATGACTTCGAAA      360
A016R      AGGAAGACATGAATGTCAAGTTTTTCAATAGCAACAAAAAGAAACGAGATGACTTCGAAA      360
A017R      AGGAAGACATGAATGTCAAGTTTTTCAATAGCAACAAAAAGAAACGAGATGACTTCGAAA      357
A018R      AGGAAGACATGAATGTCAAGTTTTTCAATAGCAACAAAAAGAAACGAGATGACTTCGAAA      356
N006R      AGGAAGACATGAATGTCAAGTTTTTCAATAGCAACAAAAAGAAACGAGATGACTTCGAAA      357
N007R      AGGAAGACATGAATGTCAAGTTTTTCAATAGCAACAAAAAGAAACGAGATGACTTCGAAA      357
N009R      AGGAAGACATGAATGTCAAGTTTTTCAATAGCAACAAAAAGAAACGAGATGACTTCGAAA      356
N010R      AGGAAGACATGAATGTCAAGTTTTTCAATAGCAACAAAAAGAAACGAGATGACTTCGAAA      359
A004R      AGGAAGACATGAATGTCAAGTTTTTCAATAGCAACAAAAAGAAACGAGATGACTTCGAAA      359
A009R      AGGAAGACATGAATGTCAAGTTTTTCAATAGCAACAAAAAGAAACGAGATGACTTCGAAA      360
A011R      AGGAAGACATGAATGTCAAGTTTTTCAATAGCAACAAAAAGAAACGAGATGACTTCGAAA      360
A014R      AGGAAGACATGAATGTCAAGTTTTTCAATAGCAACAAAAAGAAACGAGATGACTTCGAAA      358
N001R      AGGAAGACATGAATGTCAAGTTTTTCAATAGCAACAAAAAGAAACGAGATGACTTCGAAA      358
           ************************************************************

N001F      AGCTGACTAATTATTCGGTAACTGACTTGAATGTCCAACGCAAAGCAATACATGAACTCA      369
A009F      AGCTGACTAATTATTCGGTAACTGACTTGAATGTCCAACGCAAAGCAATACATGAACTCA      369
A002F      AGCTGACTAATTATTCGGTAACTGACTTGAATGTCCAACGCAAAGCAATACATGAACTCA      377
A004F      AGCTGACTAATTATTCGGTAACTGACTTGAATGTCCAACGCAAAGCAATACATGAACTCA      377
A008F      AGCTGACTAATTATTCGGTAACTGACTTGAATGTCCAACGCAAAGCAATACATGAACTCA      377
A011F      AGCTGACTAATTATTCGGTAACTGACTTGAATGTCCAACGCAAAGCAATACATGAACTCA      377
A014F      AGCTGACTAATTATTCGGTAACTGACTTGAATGTCCAACGCAAAGCAATACATGAACTCA      377
A015F      AGCTGACTAATTATTCGGTAACTGACTTGAATGTCCAACGCAAAGCAATACATGAACTCA      377
A016F      AGCTGACTAATTATTCGGTAACTGACTTGAATGTCCAACGCAAAGCAATACATGAACTCA      377
A017F      AGCTGACTAATTATTCGGTAACTGACTTGAATGTCCAACGCAAAGCAATACATGAACTCA      377
A018F      AGCTGACTAATTATTCGGTAACTGACTTGAATGTCCAACGCAAAGCAATACATGAACTCA      377
N006F      AGCTGACTAATTATTCGGTAACTGACTTGAATGTCCAACGCAAAGCAATACATGAACTCA      377
N007F      AGCTGACTAATTATTCGGTAACTGACTTGAATGTCCAACGCAAAGCAATACATGAACTCA      377
N009F      AGCTGACTAATTATTCGGTAACTGACTTGAATGTCCAACGCAAAGCAATACATGAACTCA      377
N010F      AGCTGACTAATTATTCGGTAACTGACTTGAATGTCCAACGCAAAGCAATACATGAACTCA      377
A002R      AGCTGACTAATTATTCGGTAACTGACTTGAATGTCCAACGCAAAGCAATACATGAACTCA      416
A008R      AGCTGACTAATTATTCGGTAACTGACTTGAATGTCCAACGCAAAGCAATACATGAACTCA      417
A015R      AGCTGACTAATTATTCGGTAACTGACTTGAATGTCCAACGCAAAGCAATACATGAACTCA      420
A016R      AGCTGACTAATTATTCGGTAACTGACTTGAATGTCCAACGCAAAGCAATACATGAACTCA      420
A017R      AGCTGACTAATTATTCGGTAACTGACTTGAATGTCCAACGCAAAGCAATACATGAACTCA      417
A018R      AGCTGACTAATTATTCGGTAACTGACTTGAATGTCCAACGCAAAGCAATACATGAACTCA      416
N006R      AGCTGACTAATTATTCGGTAACTGACTTGAATGTCCAACGCAAAGCAATACATGAACTCA      417
N007R      AGCTGACTAATTATTCGGTAACTGACTTGAATGTCCAACGCAAAGCAATACATGAACTCA      417
N009R      AGCTGACTAATTATTCGGTAACTGACTTGAATGTCCAACGCAAAGCAATACATGAACTCA      416
N010R      AGCTGACTAATTATTCGGTAACTGACTTGAATGTCCAACGCAAAGCAATACATGAACTCA      419
```

```
A004R      AGCTGACTAATTATTCGGTAACTGACTTGAATGTCCAACGCAAAGCAATACATGAACTCA      419
A009R      AGCTGACTAATTATTCGGTAACTGACTTGAATGTCCAACGCAAAGCAATACATGAACTCA      420
A011R      AGCTGACTAATTATTCGGTAACTGACTTGAATGTCCAACGCAAAGCAATACATGAACTCA      420
A014R      AGCTGACTAATTATTCGGTAACTGACTTGAATGTCCAACGCAAAGCAATACATGAACTCA      418
N001R      AGCTGACTAATTATTCGGTAACTGACTTGAATGTCCAACGCAAAGCAATACATGAACTCA      418
           ************************************************************

N001F      TCCAAGTGATGGCTGAACTGTCGCCAGCAGCTAAAACAGGGAAGCGAAAAAGGAGTCAGA      429
A009F      TCCAAGTGATGGCTGAACTGTCGCCAGCAGCTAAAACAGGGAAGCGAAAAAGGAGTCAGA      429
A002F      TCCAAGTGATGGCTGAACTGTCGCCAGCAGCTAAAACAGGGAAGCGAAAAAGGAGTCAGA      437
A004F      TCCAAGTGATGGCTGAACTGTCGCCAGCAGCTAAAACAGGGAAGCGAAAAAGGAGTCAGA      437
A008F      TCCAAGTGATGGCTGAACTGTCGCCAGCAGCTAAAACAGGGAAGCGAAAAAGGAGTCAGA      437
A011F      TCCAAGTGATGGCTGAACTGTCGCCAGCAGCTAAAACAGGGAAGCGAAAAAGGAGTCAGA      437
A014F      TCCAAGTGATGGCTGAACTGTCGCCAGCAGCTAAAACAGGGAAGCGAAAAAGGAGTCAGA      437
A015F      TCCAAGTGATGGCTGAACTGTCGCCAGCAGCTAAAACAGGGAAGCGAAAAAGGAGTCAGA      437
A016F      TCCAAGTGATGGCTGAACTGTCGCCAGCAGCTAAAACAGGGAAGCGAAAAAGGAGTCAGA      437
A017F      TCCAAGTGATGGCTGAACTGTCGCCAGCAGCTAAAACAGGGAAGCGAAAAAGGAGTCAGA      437
A018F      TCCAAGTGATGGCTGAACTGTCGCCAGCAGCTAAAACAGGGAAGCGAAAAAGGAGTCAGA      437
N006F      TCCAAGTGATGGCTGAACTGTCGCCAGCAGCTAAAACAGGGAAGCGAAAAAGGAGTCAGA      437
N007F      TCCAAGTGATGGCTGAACTGTCGCCAGCAGCTAAAACAGGGAAGCGAAAAAGGAGTCAGA      437
N009F      TCCAAGTGATGGCTGAACTGTCGCCAGCAGCTAAAACAGGGAAGCGAAAAAGGAGTCAGA      437
N010F      TCCAAGTGATGGCTGAACTGTCGCCAGCAGCTAAAACAGGGAAGCGAAAAAGGAGTCAGA      437
A002R      TCCAAGTGATGGCTGAACTGTCGCCAGCAGCTAAAACAGGGAAGCGAAAAAGGA------      470
A008R      TCCAAGTGATGGCTGAACTGTCGCCAGCAGCTAAAACAGGGAAGCGAAAAAGGA------      471
A015R      TCCAAGTGATGGCTGAACTGTCGCCAGCAGCTAAAACAGGGAAGCGAAAAAGGA------      474
A016R      TCCAAGTGATGGCTGAACTGTCGCCAGCAGCTAAAACAGGGAAGCGAAAAAGGA------      474
A017R      TCCAAGTGATGGCTGAACTGTCGCCAGCAGCTAAAACAGGGAAGCGAAAAAGGA------      471
A018R      TCCAAGTGATGGCTGAACTGTCGCCAGCAGCTAAAACAGGGAAG----------------      460
N006R      TCCAAGTGATGGCTGAACTGTCGCCAGCAGCTAAAACAGGGAAGCGAAAAAGGA------      471
N007R      TCCAAGTGATGGCTGAACTGTCGCCAGCAGCTAAAACAGGGAAGCGAAAAAGGA------      471
N009R      TCCAAGTGATGGCTGAACTGTCGCCAGCAGCTAAAACAGGGAAGCGAAAAAGGA------      470
N010R      TCCAAGTGATGGCTGAACTGTCGCCAGCAGCTAAAACAGGGAAGCGAAAAAGGA------      473
A004R      TCCAAGTGATGGCTGAACTGTCGCCAGCAGCTAAAACAGGGAAG----------------      463
A009R      TCCAAGTGATGGCTGAACTGTCGCCAGCAGCTAAAACAGGGAAG----------------      464
A011R      TCCAAGTGATGGCTGAACTGTCGCCAGCAGCTAAAACAGGGAAG----------------      464
A014R      TCCAAGTGATGGCTGAACTGTCGCCAGCAGCTAAAACAGGGAAG----------------      462
N001R      TCCAAGTGATGGCTGAACTGTCGCCAGCAGCTAAAACAGGGAAG----------------      462
           ********************************************

N001F      TGCTGTTTCGAGGTCGAAGAGCATCCCA--- 457
A009F      TGCTGTTTCGAGGTCGAAGAGCATCCCA--- 457
A002F      TGCTGTTTCGAGGTCGAAGAGCATCCC---- 464
A004F      TGCTGTTTCGAGGTCGAAGAGCATCCCA--- 465
A008F      TGCTGTTTCGAGGTCGAAGAGCATCCCA--- 465
A011F      TGCTGTTTCGAGGTCGAAGAGCATCCCAGTA 468
A014F      TGCTGTTTCGAGGTCGAAGAGCATCCCA--- 465
A015F      TGCTGTTTCGAGGTCGAAGAGCATCCCA--- 465
A016F      TGCTGTTTCGAGGTCGAAGAGCATCCCA--- 465
A017F      TGCTGTTTCGAGGTCGAAGAGCATCCCA--- 465
A018F      TGCTGTTTCGAGGTCGAAGAGCATCCCA--- 465
N006F      TGCTGTTTCGAGGTCGAAGAGCATCCCA--- 465
N007F      TGCTGTTTCGAGGTCGAAGAGCATCCCA--- 465
N009F      TGCTGTTTCGAGGTCGAAGAGCATCCCA--- 465
N010F      TGCTGTTTCGAGGTCGAAGAGCATCCCA--- 465
A002R      ------------------------------- 470
A008R      ------------------------------- 471
A015R      ------------------------------- 474
A016R      ------------------------------- 474
A017R      ------------------------------- 471
A018R      ------------------------------- 460
N006R      ------------------------------- 471
N007R      ------------------------------- 471
N009R      ------------------------------- 470
```

```
N010R      ------------------------------- 473
A004R      ------------------------------- 463
A009R      ------------------------------- 464
A011R      ------------------------------- 464
A014R      ------------------------------- 462
N001R      ------------------------------- 462
```

SET 3

```
A021F      -------------------------------------------GCTCTGCATCGTTTTGG     17
A022F      -------------------------------------------GCTCTGCATCGTTTTGG     17
A025F      -------------------------------------------GCTCTGCATCGTTTTGG     17
A026F      -------------------------------------------GCTCTGCATCGTTTTGG     17
A027F      -------------------------------------------GCTCTGCATCGTTTTGG     17
N015F      -------------------------------------------GCTCTGCATCGTTTTGG     17
N016F      -------------------------------------------GCTCTGCATCGTTTTGG     17
N018F      -------------------------------------------GCTCTGCATCGTTTTGG     17
N019F      -------------------------------------------GCTCTGCATCGTTTTGG     17
N021F      -------------------------------------------GCTCTGCATCGTTTTGG     17
N018R      TCTCGGAAACGATGAAATATACAAGTTATATCTTGGCTTTTCAGCTCTGCATCGTTTTGG     60
N016R      TCTCGGAAACGATGAAATATACAAGTTATATCTTGGCTTTTCAGCTCTGCATCGTTTTGG     60
N015R      TCTCGGAAACGATGAAATATACAAGTTATATCTTGGCTTTTCAGCTCTGCATCGTTTTGG     60
A021R      TCTCGGAAACGATGAAATATACAAGTTATATCTTGGCTTTTCAGCTCTGCATCGTTTTGG     60
A022R      TCTCGGAAACGATGAAATATACAAGTTATATCTTGGCTTTTCAGCTCTGCATCGTTTTGG     60
A025R      TCTCGGAAACGATGAAATATACAAGTTATATCTTGGCTTTTCAGCTCTGCATCGTTTTGG     60
A026R      TCTCGGAAACGATGAAATATACAAGTTATATCTTGGCTTTTCAGCTCTGCATCGTTTTGG     60
A027R      TCTCGGAAACGATGAAATATACAAGTTATATCTTGGCTTTTCAGCTCTGCATCGTTTTGG     60
N019R      TCTCGGAAACGATGAAATATACAAGTTATATCTTGGCTTTTCAGCTCTGCATCGTTTTGG     60
N021R      TCTCGGAAACGATGAAATATACAAGTTATATCTTGGCTTTTCAGCTCTGCATCGTTTTGG     60
                                                      *****************

A021F      GTTCTCTTGGCTGTTACTGCCAGGACCCATATGTAAAAGAAGCAGAAAACCTTAAGAAAT     77
A022F      GTTCTCTTGGCTGTTACTGCCAGGACCCATATGTAAAAGAAGCAGAAAACCTTAAGAAAT     77
A025F      GTTCTCTTGGCTGTTACTGCCAGGACCCATATGTAAAAGAAGCAGAAAACCTTAAGAAAT     77
A026F      GTTCTCTTGGCTGTTACTGCCAGGACCCATATGTAAAAGAAGCAGAAAACCTTAAGAAAT     77
A027F      GTTCTCTTGGCTGTTACTGCCAGGACCCATATGTAAAAGAAGCAGAAAACCTTAAGAAAT     77
N015F      GTTCTCTTGGCTGTTACTGCCAGGACCCATATGTAAAAGAAGCAGAAAACCTTAAGAAAT     77
N016F      GTTCTCTTGGCTGTTACTGCCAGGACCCATATGTAAAAGAAGCAGAAAACCTTAAGAAAT     77
N018F      GTTCTCTTGGCTGTTACTGCCAGGACCCATATGTAAAAGAAGCAGAAAACCTTAAGAAAT     77
N019F      GTTCTCTTGGCTGTTACTGCCAGGACCCATATGTAAAAGAAGCAGAAAACCTTAAGAAAT     77
N021F      GTTCTCTTGGCTGTTACTGCCAGGACCCATATGTAAAAGAAGCAGAAAACCTTAAGAAAT     77
N018R      GTTCTCTTGGCTGTTACTGCCAGGACCCATATGTAAAAGAAGCAGAAAACCTTAAGAAAT     120
N016R      GTTCTCTTGGCTGTTACTGCCAGGACCCATATGTAAAAGAAGCAGAAAACCTTAAGAAAT     120
N015R      GTTCTCTTGGCTGTTACTGCCAGGACCCATATGTAAAAGAAGCAGAAAACCTTAAGAAAT     120
A021R      GTTCTCTTGGCTGTTACTGCCAGGACCCATATGTAAAAGAAGCAGAAAACCTTAAGAAAT     120
A022R      GTTCTCTTGGCTGTTACTGCCAGGACCCATATGTAAAAGAAGCAGAAAACCTTAAGAAAT     120
A025R      GTTCTCTTGGCTGTTACTGCCAGGACCCATATGTAAAAGAAGCAGAAAACCTTAAGAAAT     120
A026R      GTTCTCTTGGCTGTTACTGCCAGGACCCATATGTAAAAGAAGCAGAAAACCTTAAGAAAT     120
A027R      GTTCTCTTGGCTGTTACTGCCAGGACCCATATGTAAAAGAAGCAGAAAACCTTAAGAAAT     120
N019R      GTTCTCTTGGCTGTTACTGCCAGGACCCATATGTAAAAGAAGCAGAAAACCTTAAGAAAT     120
N021R      GTTCTCTTGGCTGTTACTGCCAGGACCCATATGTAAAAGAAGCAGAAAACCTTAAGAAAT     120
           ************************************************************

A021F      ATTTTAATGCAGGTCATTCAGATGTAGCGGATAATGGAACTCTTTTCTTAGGCATTTTGA     137
A022F      ATTTTAATGCAGGTCATTCAGATGTAGCGGATAATGGAACTCTTTTCTTAGGCATTTTGA     137
A025F      ATTTTAATGCAGGTCATTCAGATGTAGCGGATAATGGAACTCTTTTCTTAGGCATTTTGA     137
A026F      ATTTTAATGCAGGTCATTCAGATGTAGCGGATAATGGAACTCTTTTCTTAGGCATTTTGA     137
A027F      ATTTTAATGCAGGTCATTCAGATGTAGCGGATAATGGAACTCTTTTCTTAGGCATTTTGA     137
N015F      ATTTTAATGCAGGTCATTCAGATGTAGCGGATAATGGAACTCTTTTCTTAGGCATTTTGA     137
N016F      ATTTTAATGCAGGTCATTCAGATGTAGCGGATAATGGAACTCTTTTCTTAGGCATTTTGA     137
N018F      ATTTTAATGCAGGTCATTCAGATGTAGCGGATAATGGAACTCTTTTCTTAGGCATTTTGA     137
N019F      ATTTTAATGCAGGTCATTCAGATGTAGCGGATAATGGAACTCTTTTCTTAGGCATTTTGA     137
```

```
N021F     ATTTTAATGCAGGTCATTCAGATGTAGCGGATAATGGAACTCTTTTCTTAGGCATTTTGA     137
N018R     ATTTTAATGCAGGTCATTCAGATGTAGCGGATAATGGAACTCTTTTCTTAGGCATTTTGA     180
N016R     ATTTTAATGCAGGTCATTCAGATGTAGCGGATAATGGAACTCTTTTCTTAGGCATTTTGA     180
N015R     ATTTTAATGCAGGTCATTCAGATGTAGCGGATAATGGAACTCTTTTCTTAGGCATTTTGA     180
A021R     ATTTTAATGCAGGTCATTCAGATGTAGCGGATAATGGAACTCTTTTCTTAGGCATTTTGA     180
A022R     ATTTTAATGCAGGTCATTCAGATGTAGCGGATAATGGAACTCTTTTCTTAGGCATTTTGA     180
A025R     ATTTTAATGCAGGTCATTCAGATGTAGCGGATAATGGAACTCTTTTCTTAGGCATTTTGA     180
A026R     ATTTTAATGCAGGTCATTCAGATGTAGCGGATAATGGAACTCTTTTCTTAGGCATTTTGA     180
A027R     ATTTTAATGCAGGTCATTCAGATGTAGCGGATAATGGAACTCTTTTCTTAGGCATTTTGA     180
N019R     ATTTTAATGCAGGTCATTCAGATGTAGCGGATAATGGAACTCTTTTCTTAGGCATTTTGA     180
N021R     ATTTTAATGCAGGTCATTCAGATGTAGCGGATAATGGAACTCTTTTCTTAGGCATTTTGA     180
          ************************************************************

A021F     AGAATTGGAAAGAGGAGAGTGACAGAAAAATAATGCAGAGCCAAATTGTCTCCTTTTACT     197
A022F     AGAATTGGAAAGAGGAGAGTGACAGAAAAATAATGCAGAGCCAAATTGTCTCCTTTTACT     197
A025F     AGAATTGGAAAGAGGAGAGTGACAGAAAAATAATGCAGAGCCAAATTGTCTCCTTTTACT     197
A026F     AGAATTGGAAAGAGGAGAGTGACAGAAAAATAATGCAGAGCCAAATTGTCTCCTTTTACT     197
A027F     AGAATTGGAAAGAGGAGAGTGACAGAAAAATAATGCAGAGCCAAATTGTCTCCTTTTACT     197
N015F     AGAATTGGAAAGAGGAGAGTGACAGAAAAATAATGCAGAGCCAAATTGTCTCCTTTTACT     197
N016F     AGAATTGGAAAGAGGAGAGTGACAGAAAAATAATGCAGAGCCAAATTGTCTCCTTTTACT     197
N018F     AGAATTGGAAAGAGGAGAGTGACAGAAAAATAATGCAGAGCCAAATTGTCTCCTTTTACT     197
N019F     AGAATTGGAAAGAGGAGAGTGACAGAAAAATAATGCAGAGCCAAATTGTCTCCTTTTACT     197
N021F     AGAATTGGAAAGAGGAGAGTGACAGAAAAATAATGCAGAGCCAAATTGTCTCCTTTTACT     197
N018R     AGAATTGGAAAGAGGAGAGTGACAGAAAAATAATGCAGAGCCAAATTGTCTCCTTTTACT     240
N016R     AGAATTGGAAAGAGGAGAGTGACAGAAAAATAATGCAGAGCCAAATTGTCTCCTTTTACT     240
N015R     AGAATTGGAAAGAGGAGAGTGACAGAAAAATAATGCAGAGCCAAATTGTCTCCTTTTACT     240
A021R     AGAATTGGAAAGAGGAGAGTGACAGAAAAATAATGCAGAGCCAAATTGTCTCCTTTTACT     240
A022R     AGAATTGGAAAGAGGAGAGTGACAGAAAAATAATGCAGAGCCAAATTGTCTCCTTTTACT     240
A025R     AGAATTGGAAAGAGGAGAGTGACAGAAAAATAATGCAGAGCCAAATTGTCTCCTTTTACT     240
A026R     AGAATTGGAAAGAGGAGAGTGACAGAAAAATAATGCAGAGCCAAATTGTCTCCTTTTACT     240
A027R     AGAATTGGAAAGAGGAGAGTGACAGAAAAATAATGCAGAGCCAAATTGTCTCCTTTTACT     240
N019R     AGAATTGGAAAGAGGAGAGTGACAGAAAAATAATGCAGAGCCAAATTGTCTCCTTTTACT     240
N021R     AGAATTGGAAAGAGGAGAGTGACAGAAAAATAATGCAGAGCCAAATTGTCTCCTTTTACT     240
          ************************************************************

A021F     TCAAACTTTTTAAAAACTTTAAAGATGACCAGAGCATCCAAAAGAGTGTGGAGACCATCA     257
A022F     TCAAACTTTTTAAAAACTTTAAAGATGACCAGAGCATCCAAAAGAGTGTGGAGACCATCA     257
A025F     TCAAACTTTTTAAAAACTTTAAAGATGACCAGAGCATCCAAAAGAGTGTGGAGACCATCA     257
A026F     TCAAACTTTTTAAAAACTTTAAAGATGACCAGAGCATCCAAAAGAGTGTGGAGACCATCA     257
A027F     TCAAACTTTTTAAAAACTTTAAAGATGACCAGAGCATCCAAAAGAGTGTGGAGACCATCA     257
N015F     TCAAACTTTTTAAAAACTTTAAAGATGACCAGAGCATCCAAAAGAGTGTGGAGACCATCA     257
N016F     TCAAACTTTTTAAAAACTTTAAAGATGACCAGAGCATCCAAAAGAGTGTGGAGACCATCA     257
N018F     TCAAACTTTTTAAAAACTTTAAAGATGACCAGAGCATCCAAAAGAGTGTGGAGACCATCA     257
N019F     TCAAACTTTTTAAAAACTTTAAAGATGACCAGAGCATCCAAAAGAGTGTGGAGACCATCA     257
N021F     TCAAACTTTTTAAAAACTTTAAAGATGACCAGAGCATCCAAAAGAGTGTGGAGACCATCA     257
N018R     TCAAACTTTTTAAAAACTTTAAAGATGACCAGAGCATCCAAAAGAGTGTGGAGACCATCA     300
N016R     TCAAACTTTTTAAAAACTTTAAAGATGACCAGAGCATCCAAAAGAGTGTGGAGACCATCA     300
N015R     TCAAACTTTTTAAAAACTTTAAAGATGACCAGAGCATCCAAAAGAGTGTGGAGACCATCA     300
A021R     TCAAACTTTTTAAAAACTTTAAAGATGACCAGAGCATCCAAAAGAGTGTGGAGACCATCA     300
A022R     TCAAACTTTTTAAAAACTTTAAAGATGACCAGAGCATCCAAAAGAGTGTGGAGACCATCA     300
A025R     TCAAACTTTTTAAAAACTTTAAAGATGACCAGAGCATCCAAAAGAGTGTGGAGACCATCA     300
A026R     TCAAACTTTTTAAAAACTTTAAAGATGACCAGAGCATCCAAAAGAGTGTGGAGACCATCA     300
A027R     TCAAACTTTTTAAAAACTTTAAAGATGACCAGAGCATCCAAAAGAGTGTGGAGACCATCA     300
N019R     TCAAACTTTTTAAAAACTTTAAAGATGACCAGAGCATCCAAAAGAGTGTGGAGACCATCA     300
N021R     TCAAACTTTTTAAAAACTTTAAAGATGACCAGAGCATCCAAAAGAGTGTGGAGACCATCA     300
          ************************************************************

A021F     AGGAAGACATGAATGTCAAGTTTTTCAATAGCAACAAAAAGAAACGAGATGACTTCGAAA     317
A022F     AGGAAGACATGAATGTCAAGTTTTTCAATAGCAACAAAAAGAAACGAGATGACTTCGAAA     317
A025F     AGGAAGACATGAATGTCAAGTTTTTCAATAGCAACAAAAAGAAACGAGATGACTTCGAAA     317
A026F     AGGAAGACATGAATGTCAAGTTTTTCAATAGCAACAAAAAGAAACGAGATGACTTCGAAA     317
A027F     AGGAAGACATGAATGTCAAGTTTTTCAATAGCAACAAAAAGAAACGAGATGACTTCGAAA     317
N015F     AGGAAGACATGAATGTCAAGTTTTTCAATAGCAACAAAAAGAAACGAGATGACTTCGAAA     317
```

```
N016F     AGGAAGACATGAATGTCAAGTTTTTCAATAGCAACAAAAAGAAACGAGATGACTTCGAAA     317
N018F     AGGAAGACATGAATGTCAAGTTTTTCAATAGCAACAAAAAGAAACGAGATGACTTCGAAA     317
N019F     AGGAAGACATGAATGTCAAGTTTTTCAATAGCAACAAAAAGAAACGAGATGACTTCGAAA     317
N021F     AGGAAGACATGAATGTCAAGTTTTTCAATAGCAACAAAAAGAAACGAGATGACTTCGAAA     317
N018R     AGGAAGACATGAATGTCAAGTTTTTCAATAGCAACAAAAAGAAACGAGATGACTTCGAAA     360
N016R     AGGAAGACATGAATGTCAAGTTTTTCAATAGCAACAAAAAGAAACGAGATGACTTCGAAA     360
N015R     AGGAAGACATGAATGTCAAGTTTTTCAATAGCAACAAAAAGAAACGAGATGACTTCGAAA     360
A021R     AGGAAGACATGAATGTCAAGTTTTTCAATAGCAACAAAAAGAAACGAGATGACTTCGAAA     360
A022R     AGGAAGACATGAATGTCAAGTTTTTCAATAGCAACAAAAAGAAACGAGATGACTTCGAAA     360
A025R     AGGAAGACATGAATGTCAAGTTTTTCAATAGCAACAAAAAGAAACGAGATGACTTCGAAA     360
A026R     AGGAAGACATGAATGTCAAGTTTTTCAATAGCAACAAAAAGAAACGAGATGACTTCGAAA     360
A027R     AGGAAGACATGAATGTCAAGTTTTTCAATAGCAACAAAAAGAAACGAGATGACTTCGAAA     360
N019R     AGGAAGACATGAATGTCAAGTTTTTCAATAGCAACAAAAAGAAACGAGATGACTTCGAAA     360
N021R     AGGAAGACATGAATGTCAAGTTTTTCAATAGCAACAAAAAGAAACGAGATGACTTCGAAA     360
          ************************************************************

A021F     AGCTGACTAATTATTCGGTAACTGACTTGAATGTCCAACGCAAAGCAATACATGAACTCA     377
A022F     AGCTGACTAATTATTCGGTAACTGACTTGAATGTCCAACGCAAAGCAATACATGAACTCA     377
A025F     AGCTGACTAATTATTCGGTAACTGACTTGAATGTCCAACGCAAAGCAATACATGAACTCA     377
A026F     AGCTGACTAATTATTCGGTAACTGACTTGAATGTCCAACGCAAAGCAATACATGAACTCA     377
A027F     AGCTGACTAATTATTCGGTAACTGACTTGAATGTCCAACGCAAAGCAATACATGAACTCA     377
N015F     AGCTGACTAATTATTCGGTAACTGACTTGAATGTCCAACGCAAAGCAATACATGAACTCA     377
N016F     AGCTGACTAATTATTCGGTAACTGACTTGAATGTCCAACGCAAAGCAATACATGAACTCA     377
N018F     AGCTGACTAATTATTCGGTAACTGACTTGAATGTCCAACGCAAAGCAATACATGAACTCA     377
N019F     AGCTGACTAATTATTCGGTAACTGACTTGAATGTCCAACGCAAAGCAATACATGAACTCA     377
N021F     AGCTGACTAATTATTCGGTAACTGACTTGAATGTCCAACGCAAAGCAATACATGAACTCA     377
N018R     AGCTGACTAATTATTCGGTAACTGACTTGAATGTCCAACGCAAAGCAATACATGAACTCA     420
N016R     AGCTGACTAATTATTCGGTAACTGACTTGAATGTCCAACGCAAAGCAATACATGAACTCA     420
N015R     AGCTGACTAATTATTCGGTAACTGACTTGAATGTCCAACGCAAAGCAATACATGAACTCA     420
A021R     AGCTGACTAATTATTCGGTAACTGACTTGAATGTCCAACGCAAAGCAATACATGAACTCA     420
A022R     AGCTGACTAATTATTCGGTAACTGACTTGAATGTCCAACGCAAAGCAATACATGAACTCA     420
A025R     AGCTGACTAATTATTCGGTAACTGACTTGAATGTCCAACGCAAAGCAATACATGAACTCA     420
A026R     AGCTGACTAATTATTCGGTAACTGACTTGAATGTCCAACGCAAAGCAATACATGAACTCA     420
A027R     AGCTGACTAATTATTCGGTAACTGACTTGAATGTCCAACGCAAAGCAATACATGAACTCA     420
N019R     AGCTGACTAATTATTCGGTAACTGACTTGAATGTCCAACGCAAAGCAATACATGAACTCA     420
N021R     AGCTGACTAATTATTCGGTAACTGACTTGAATGTCCAACGCAAAGCAATACATGAACTCA     420
          ************************************************************

A021F     TCCAAGTGATGGCTGAACTGTCGCCAGCAGCTAAAACAGGGAAGCGAAAAAGGAGTCAGA     437
A022F     TCCAAGTGATGGCTGAACTGTCGCCAGCAGCTAAAACAGGGAAGCGAAAAAGGAGTCAGA     437
A025F     TCCAAGTGATGGCTGAACTGTCGCCAGCAGCTAAAACAGGGAAGCGAAAAAGGAGTCAGA     437
A026F     TCCAAGTGATGGCTGAACTGTCGCCAGCAGCTAAAACAGGGAAGCGAAAAAGGAGTCAGA     437
A027F     TCCAAGTGATGGCTGAACTGTCGCCAGCAGCTAAAACAGGGAAGCGAAAAAGGAGTCAGA     437
N015F     TCCAAGTGATGGCTGAACTGTCGCCAGCAGCTAAAACAGGGAAGCGAAAAAGGAGTCAGA     437
N016F     TCCAAGTGATGGCTGAACTGTCGCCAGCAGCTAAAACAGGGAAGCGAAAAAGGAGTCAGA     437
N018F     TCCAAGTGATGGCTGAACTGTCGCCAGCAGCTAAAACAGGGAAGCGAAAAAGGAGTCAGA     437
N019F     TCCAAGTGATGGCTGAACTGTCGCCAGCAGCTAAAACAGGGAAGCGAAAAAGGAGTCAGA     437
N021F     TCCAAGTGATGGCTGAACTGTCGCCAGCAGCTAAAACAGGGAAGCGAAAAAGGAGTCAGA     437
N018R     TCCAAGTGATGGCTGAACTGTCGCCAGCAGCTAAAACAGGGAAG----------------     464
N016R     TCCAAGTGATGGCTGAACTGTCGCCAGCAGCTAAAACAGGGAAG----------------     464
N015R     TCCAAGTGATGGCTGAACTGTCGCCAGCAGCTAAAACAGGGAAGCGAAAAAGGA------     474
A021R     TCCAAGTGATGGCTGAACTGTCGCCAGCAGCTAAAACA----------------------     458
A022R     TCCAAGTGATGGCTGAACTGTCGCCAGCAGCTAAAACA----------------------     458
A025R     TCCAAGTGATGGCTGAACTGTCGCCAGCAGCTAAAACAGGAAGCGAAAA-----------     469
A026R     TCCAAGTGATGGCTGAACTGTCGCCAGCAGCTAAAACAGGAAG-----------------     463
A027R     TCCAAGTGATGGCTGAACTGTCGCCAGCAGCTAAAACA----------------------     458
N019R     TCCAAGTGATGGCTGAACTGTCGCCAGCAGCTAAAACA----------------------     458
N021R     TCCAAGTGATGGCTGAACTGTCGCCAGCAGCTAAAACA----------------------     458
          **************************************

A021F     TGCTGTTTCGAGGTCGAAGAGCATCCCAG-- 466
A022F     TGCTGTTTCGAGGTCGAAGAGCATCCCAGTA 468
A025F     TGCTGTTTCGAGGTCGAAGAGCATCCCAGTA 468
```

```
A026F      TGCTGTTTCGAGGTCGAAGAGCATCCCAGT- 467
A027F      TGCTGTTTCGAGGTCGAAGAGCATCCCAG-- 466
N015F      TGCTGTTTCGAGGTCGAAGAGCATCCCAGTA 468
N016F      TGCTGTTTCGAGGTCGAAGAGCATCCCAGTA 468
N018F      TGCTGTTTCGAGGTCGAAGAGCATCCCAGTA 468
N019F      TGCTGTTTCGAGGTCGAAGAGCATCCCA--- 465
N021F      TGCTGTTTCGAGGTCGAAGAGCATCCCAGT- 467
N018R      ------------------------------- 464
N016R      ------------------------------- 464
N015R      ------------------------------- 474
A021R      ------------------------------- 458
A022R      ------------------------------- 458
A025R      ------------------------------- 469
A026R      ------------------------------- 463
A027R      ------------------------------- 458
N019R      ------------------------------- 458
N021R      ------------------------------- 458
```
